# Food sensing controls *C. elegans* reproductive behavior by neuromodulatory disinhibition

**DOI:** 10.1126/sciadv.adu5829

**Published:** 2025-04-16

**Authors:** Yen-Chih Chen, Kara E. Zang, Hassan Ahamed, Niels Ringstad

**Affiliations:** Department of Cell Biology and Neuroscience Institute, New York University School of Medicine, New York, NY 10016, USA.

## Abstract

Like many organisms, the roundworm *Caenorhabditis elegans* incorporates an assessment of environmental quality into its reproductive strategy. *C. elegans* hermaphrodites release fertilized eggs into food-rich environments but retain them in the absence of food. Here, we report the discovery of a neural circuit required for the modulation of reproductive behavior by food sensing. A mutation that electrically silences the AVK interneurons uncouples egg laying from detection of environmental food cues. We find that AVK activity inhibits egg laying, and AVKs themselves are inhibited by dopamine released from food-sensing neurons. AVKs express a large number of structurally and functionally diverse neuropeptides. Coordination of food-sensing and reproductive behavior requires a subset of AVK neuropeptides that converge on a small ensemble of premotor neurons that coexpress their cognate receptors. Modulation of *C. elegans* reproductive behavior, therefore, requires a cascade of neuromodulatory signals that uses disinhibition and combinatorial neuropeptide signals to activate reproductive behavior when food is sensed.

## Teaser

*C. elegans* reproductive behavior is governed by neuromodulators that couple food-sensing neurons to the egg-laying system.

## INTRODUCTION

Decisions about when and where to reproduce affect both parental fitness and the fitness of offspring. Accordingly, animals from diverse phyla adjust their reproductive strategies in response to perceived or anticipated changes in the environment, including the free-living roundworm *Caenorhabditis elegans* (*1*–*3*). One key environmental factor that controls reproductive behaviors is the availability of food resources. *C. elegans* hermaphrodites deposit fertilized eggs in patches of the bacteria on which they feed. In the absence of nutritive bacteria, hermaphrodites retain their eggs in utero (*4*, *5*). The influence of food availability on *C. elegans* egg-laying behavior is so strong that under conditions of prolonged food deprivation, *C. elegans* mothers will retain eggs until they hatch internally, often killing the mother (*6*).

Because it is compact and experimentally accessible, the neuromuscular circuit that drives *C. elegans* egg laying is well suited to the study of molecular mechanisms that regulate reproductive behavior. The *C. elegans* egg-laying system comprises 16 muscles that either apply compressive force to the uterus or dilate the vulva, four motor neurons that innervate egg-laying muscles, and a pair of neuroendocrine cells at the junction between the uterus and the vulva (*7*). Two of the four motor neurons—VC4 and VC5—are primarily cholinergic and play a modulatory role in egg laying (*8*–*11*). The other two motor neurons are the serotonergic hermaphrodite-specific neurons (HSNs), which are indispensable for egg-laying behavior (*5*, *12*, *13*). UV1 neuroendocrine cells at the interface of the uterus and the vulva are stretch sensors that release the inhibitory monoamine transmitter tyramine, which dampens the egg-laying system after an egg-laying event (*14*–*16*). In addition to small-molecule transmitters, motor neurons and neuroendocrine cells release neuropeptides that enhance the effects of neurotransmitters on their cellular targets (*11*, *17*).

While much is known about the core components of the egg-laying system and the neurochemical signals that they use, less is known about how the egg-laying system is modulated. Control of reproductive behavior by food sensing has proved particularly challenging to study because of the complexity and diversity of food-sensing mechanisms used by *C. elegans*. The bacteria on which *C. elegans* feed produce a complex suite of metabolites that activate a large number of gustatory and olfactory neurons (*18*, *19*). Bacteria are also detected by tactile food-sensing neurons as animals forage into patches of nutritive bacteria (*20*–*22*) and by interoceptive neurons that sense bacteria in the pharynx after they have been ingested (*23*).

One chemosensory modality that controls *C. elegans* egg-laying behavior is mediated by the BAG neurons, which detect changes in the levels of the respiratory gases O_2_ and CO_2_ caused by microbial respiration (*24*, *25*). BAG neurons release neuropeptides that potently inhibit HSN motor neurons via the G_i/o_α-coupled receptor EGL-6 (*26*). A gain-of-function variant of EGL-6 amplifies inhibitory neuropeptide signaling from BAG neurons and silences HSN motor neurons under conditions that normally permit egg laying (*26*). This receptor mutation offers the opportunity to use genetic analysis to delineate molecular pathways and neural circuits that modulate egg-laying behavior. Genetic modifiers of the behavior to *egl-6(gf)* mutants define genes that either (i) regulate the excitability of the core egg-laying circuit, (ii) function in the EGL-6 neuropeptide-signaling pathway, or (iii) function in a parallel pathway to modulate the egg-laying circuit.

Here, we report the discovery of a mutation that restores egg-laying behavior and HSN function to *egl-6(gf)* mutants by silencing AVK interneurons, which mediate inhibition of egg-laying behavior in parallel to the EGL-6 pathway and are required for the modulation of egg laying by food sensing. We place AVKs downstream of dopaminergic sensory neurons that are directly activated by food stimuli and that signal to AVKs via D2-type metabotropic dopamine receptors. AVK interneurons express a large number of diverse neuropeptides, and we identify a subset of three AVK neuropeptides that function together to coordinate food sensing and egg laying. Last, we show that AVK neuropeptides converge on a small number of premotor neurons, including the AIYs, that coexpress their cognate receptors and extract from a complex mix of neuropeptides a signal that is salient for control of reproductive behavior. Our study indicates that activation of *C. elegans* reproductive behavior by food stimuli requires release of the egg-laying neuromusculature from tonic inhibition that is driven by a specific combination of interneuron-derived neuropeptides. The revealed mechanism for control of *C. elegans* reproductive behavior relies upon a functional architecture of neuromodulation that we suggest is widely used in animal nervous systems.

## RESULTS

### The *n5210* mutation restores activity to the egg-laying system of mutants with excess inhibitory neuropeptide signaling

**Fig. 1.**
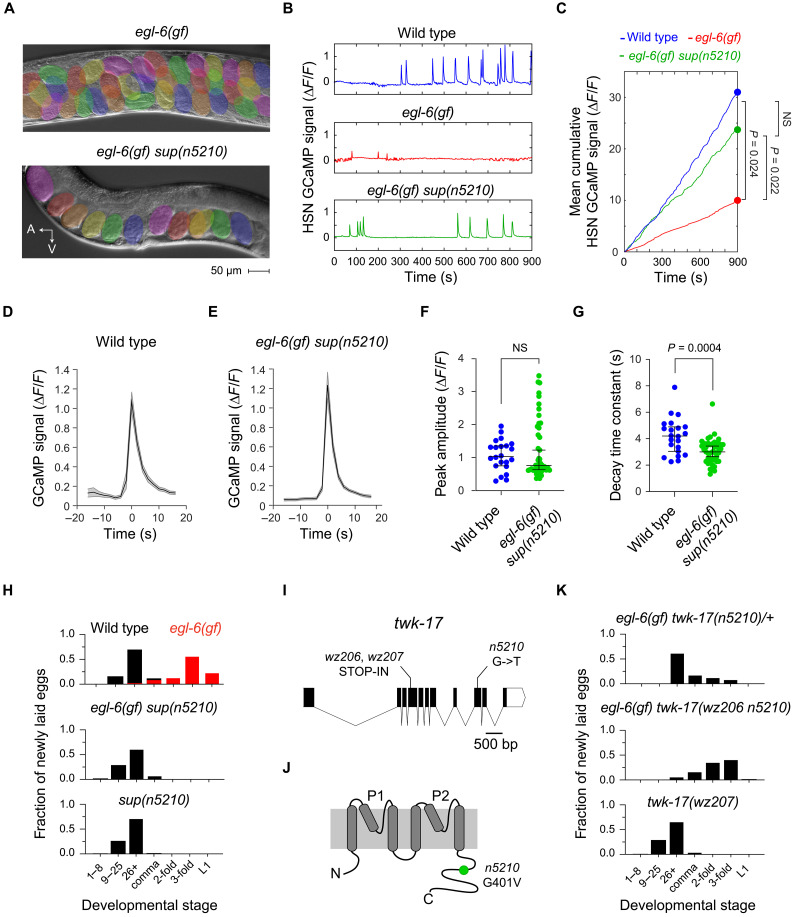
Mutation of the K_2P_ channel TWK-17 restores behavior and neuronal activity to animals whose egg-laying system receives excess inhibition. (**A**) Micrographs of *egl-6(gf)* and *egl-6(gf) sup(n5210)* animals. Eggs are pseudocolored. A, anterior; V, ventral. (**B**) Representative GCaMP signals from HSN neurons in animals. (**C**) Mean cumulative GCaMP signals from HSNs in animals. *n* = 19 to 24. (**D** and **E**) Average calcium peaks recorded from the wild type and *egl-6(gf) sup(n5210)* mutants. The shaded area indicates the SEM. (**F** and **G**) Amplitudes and decay time constants of peaks recorded from the wild type and *egl-6(gf) sup(n5210)* mutants. (**H**) Developmental stages of eggs laid by animals. *n* > 100 embryos. (**I**) *twk-17* gene model showing the *n5210* mutation and loss-of-function alleles generated by CRISPR/Cas9. bp, base pairs. (**J**) Predicted topology of the TWK-17 protein and region affected by the *n5210* mutation. (**K**) Developmental stages of eggs laid by animals. *n* > 100 embryos for each genotype. In scatterplots, error bars indicate the means ± SEM. *P* values were computed using one-way ANOVA with Tukey’s multiple comparison test (B) or with a Mann-Whitney test [(F) and (G)]. NS, not significant.

Prior studies identified a gain-of-function variant of the neuropeptide receptor EGL-6 that amplifies inhibitory neuropeptide signaling onto the HSN motor neurons of the hermaphrodite reproductive system (*26*). *egl-6(gf)* mutants bloat with unlaid eggs and are readily identified as defective for egg-laying behavior (Fig. 1A). The behavior of *egl-6(gf)* mutants can be restored by mutations that affect neuropeptide signaling and by mutations that affect HSN physiology (*27*, *28*). We therefore screened for mutations that suppress the egg-laying defect of *egl-6(gf)* mutants, reasoning that such suppressor mutations would identify genes that modulate the function of serotonergic HSN neurons. Our screen yielded the suppressor mutation *n5210*, which strongly suppressed the bloating of *egl-6(gf)* mutants with unlaid eggs (Fig. 1A). To determine the impact of the *n5210* mutation on HSN physiology, we used the Ca^2+^ indicator GCaMP6 to record the activity of HSN neurons of wild-type, *egl-6(gf)*, and *egl-6(gf) sup(n5210)* animals. Wild-type HSNs exhibited spontaneous Ca^2+^ transients that were almost completely abolished in *egl-6(gf)* mutants (Fig. 1B). The *sup(n5210)* mutation almost completely restored HSN Ca^2+^ transients to *egl-6(gf)* mutants (Fig. 1B). To quantify the Ca^2+^ activity in the HSN neurons, we calculated the cumulative GCaMP signals in the HSN cell body over time. Cumulative HSN activity in wild-type animals was similar to that in *egl-6(gf) sup(n5210)* animals, while HSN neurons in *egl-6(gf)* animals exhibited far less activity (Fig. 1C). We extracted individual calcium spikes from recordings of wild-type and *egl-6(gf) sup(n5210)* HSNs (Fig. 1, D and E) and compared their amplitudes and decay time constants. Ca^2+^ spike amplitudes were similar between wild-type and *egl-6(gf) sup(n5210)* animals (Fig. 1F), but we noted that spikes in HSNs of *egl-6(gf) sup(n5210)* animals were narrower and had faster decay time constants than spikes recorded from the wild type (Fig. 1G). Together, these findings showed that the *n5210* mutation restores function to the HSN neurons of *egl-6(gf)* mutants.

### *n5210* is a gain-of-function mutation in the two-pore domain potassium channel gene *twk-17*

To determine the effect of the *sup(n5210)* mutation on egg-laying behavior, we measured the developmental stage of newly released embryos, which reflects the amount of time an embryo spends in utero before it is released. In wild-type animals, most embryos are released before they undergo morphogenesis. By contrast, *egl-6(gf)* mutants retain embryos longer and release embryos that have developed to the threefold stage (Fig. 1H). By this measure, the egg-laying behavior of *egl-6(gf) sup(n5210)* animals was indistinguishable from that of the wild-type animals (Fig. 1H). We separated the *n5210* mutation from *egl-6(gf)* and found that on its own, *n5210* did not grossly alter how long animals retained embryos (Fig. 1H), indicating that the *n5210* mutation restored function to the egg-laying system of *egl-6(gf)* mutants without severely dysregulating its temporal dynamics.

We next sought to determine which gene was affected by the *n5210* mutation. We mapped *n5210* to the X chromosome near the *egl-6(gf)* mutation. Whole-genome sequencing revealed that the X chromosome of the suppressed strain contained only four mutations predicted to affect protein-coding sequences (fig. S1A). To identify the mutation of *n5210*, we isolated recombinants that retained the *egl-6(gf)* mutation but lost the *n5210* suppressor. One mutation, affecting the gene *twk-17*, was lost in every nonsuppressed recombinant (fig. S1B), and we designated this mutation as *n5210* (Fig. 1I). *twk-17* encodes a two-pore domain potassium (K_2P_) channel, and the *n5210* mutation is predicted to cause a glycine-to-valine mutation (Fig. 1J and fig. S2) in a cytoplasmic domain that is known to regulate the conductance of K_2P_ channels (*29*–*31*). We observed that animals heterozygous for *twk-17(n5210)* were suppressed for the effect of *egl-6(gf)* (Fig. 1K), suggesting that the *n5210* mutation might cause a gain of function in *twk-17*. To test this, we used CRISPR/Cas9 genome editing to generate loss-of-function alleles by introducing a STOP-IN cassette (*32*) into the first transmembrane domain of TWK-17 (Fig. 1I). The STOP-IN insertion by itself did not cause a gross effect on the time of egg retention (Fig. 1K), but it reverted the phenotype of suppressed *egl-6(gf) twk-17(n5210)* mutants, making them similar to unsuppressed *egl-6(gf)* mutants (Fig. 1K). These data indicate that the *n5210* suppressor mutation causes a gain of function in TWK-17, and we henceforth refer to *n5210* as *twk-17(gf)*.

### *twk-17(gf)* disrupts the regulation of egg-laying behavior by food

**Fig. 2.**
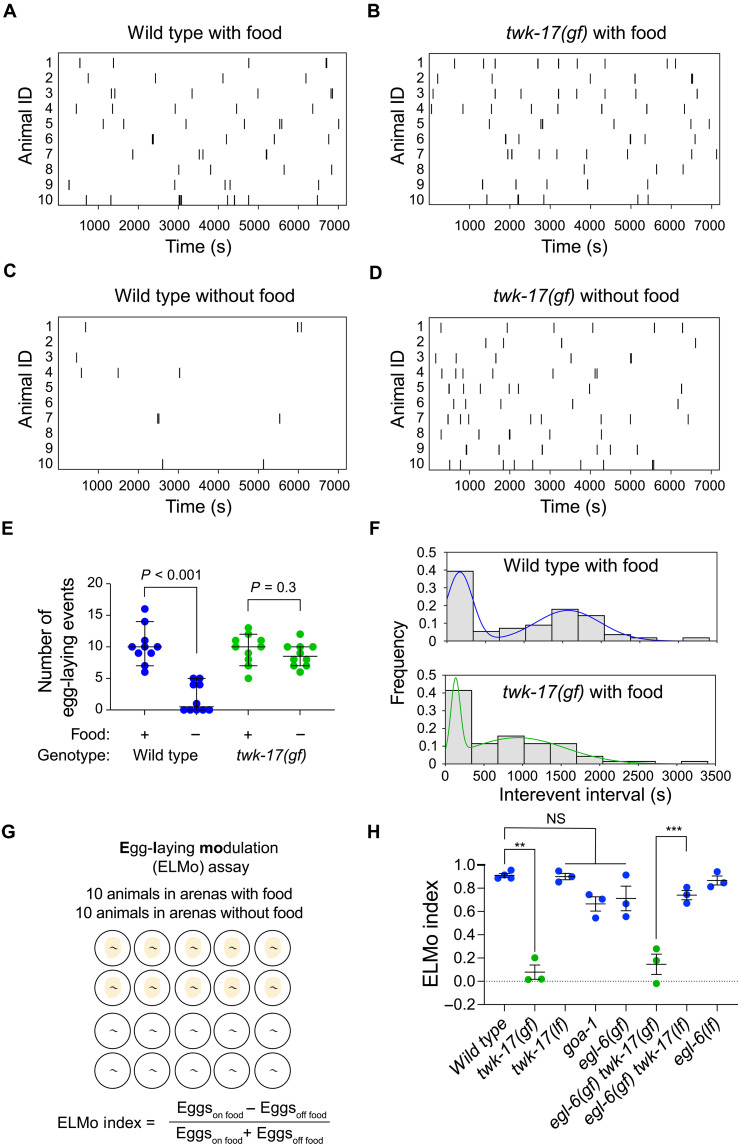
*twk-17(gf)* mutants fail to modulate egg laying in response to food cues. (**A** to **D**) Raster plots of egg-laying events detected in recordings of tracked animals in either the presence or absence of food. *n* = 10. (**E**) Scatterplots of the number of egg-laying events by wild-type and *twk-17(gf)* animals from recordings shown in (A) to (D). (**F**) Distributions of interevent intervals from recordings of the wild type and *twk-17(gf)* mutants in the presence of food. (**G**) Schematic of the ELMo assay and definition of the ELMo index. (**H**) ELMo indices of animals with the indicated genotypes. Each point is computed from one trial of 10 animals on food and 10 animals off food. In scatterplots, error bars indicate the means ± SEM. *P* values in (E) were calculated using one-way ANOVA with Tukey’s multiple comparison test. **P < 0.01; ***P < 0.001.

Food availability strongly influences egg-laying behavior (*33*, *34*). We observed that animals carrying the *twk-17(gf)* mutation had severe defects in inhibiting egg laying in the absence of food. To quantify these defects, we tracked single animals for 2 hours either in arenas with a food source, i.e., a bacterial lawn, or in arenas with no food. As expected, the egg-laying behavior of wild-type hermaphrodites was severely inhibited in the absence of food (Fig. 2, A and C). By contrast, *twk-17(gf)* mutants laid eggs at comparable rates in the presence or absence of food (Fig. 2, B and D). We observed no gross difference in the frequency of egg-laying events between the wild type and *twk-17(gf)* mutants in the presence of food (Fig. 2E), consistent with our prior observation that *twk-17* mutation does not affect egg retention (Fig. 1H). We analyzed behavior-tracking data to determine whether *twk-17(gf)* altered the timing of egg-laying events, which occur in clusters characterized by long interevent intervals that separate clusters and shorter interevent intervals within a cluster (*8*, *16*). Consistent with this model, *twk-17(gf*) mutants showed a bimodal distribution of interevent intervals, indicating that the dynamics of egg-laying behavior were grossly normal in this mutant. We noted, however, that intercluster intervals were slightly shorter in *twk-17(gf)* animals (Fig. 2F). These data indicate that *twk-17* mutation exerts a strong effect on the modulation of reproductive behavior by food sensing without markedly perturbing egg-laying behavior under basal conditions.

We streamlined our analysis of how food sensing modulates egg-laying behavior by developing an egg-laying modulation (ELMo) assay. In this assay, 10 hermaphrodites were isolated in arenas without food and 10 in arenas with food. After 2 hours, we counted the number of eggs laid by each animal and calculated the difference between eggs laid in the presence of food and in the absence of food as a fraction of the total number of eggs laid under both conditions (Fig. 2G). This metric—the ELMo index—ranges from 1 (behavior occurs only in the presence of food) through 0 (behavior occurs equally in the presence and absence of food) to −1 (behavior occurs only in the absence of food). Wild-type animals had ELMo indices close to 1, while the indices of *twk-17(gf)* animals, in either a wild-type or *egl-6(gf)* background, were close to 0 (Fig. 2H). *twk-17* loss of function did not affect the ELMo index (Fig. 2H), indicating that this defect is specifically caused by the gain of function in the channel. Furthermore, neither loss-of-function nor gain-of-function mutations of the neuropeptide receptor EGL-6 or mutation of GOA-1, which is the G_i/o_α through which EGL-6 signals (*26*), affected the ELMo index (Fig. 2H). From these data, we concluded that a gain-of-function variant of the TWK-17 potassium channel affects a mechanism that functions in parallel to the neuropeptide receptor EGL-6 to coordinate food sensing and egg laying.

### TWK-17(gf) channels disrupt the modulation of egg laying by silencing AVK interneurons

**Fig. 3.**
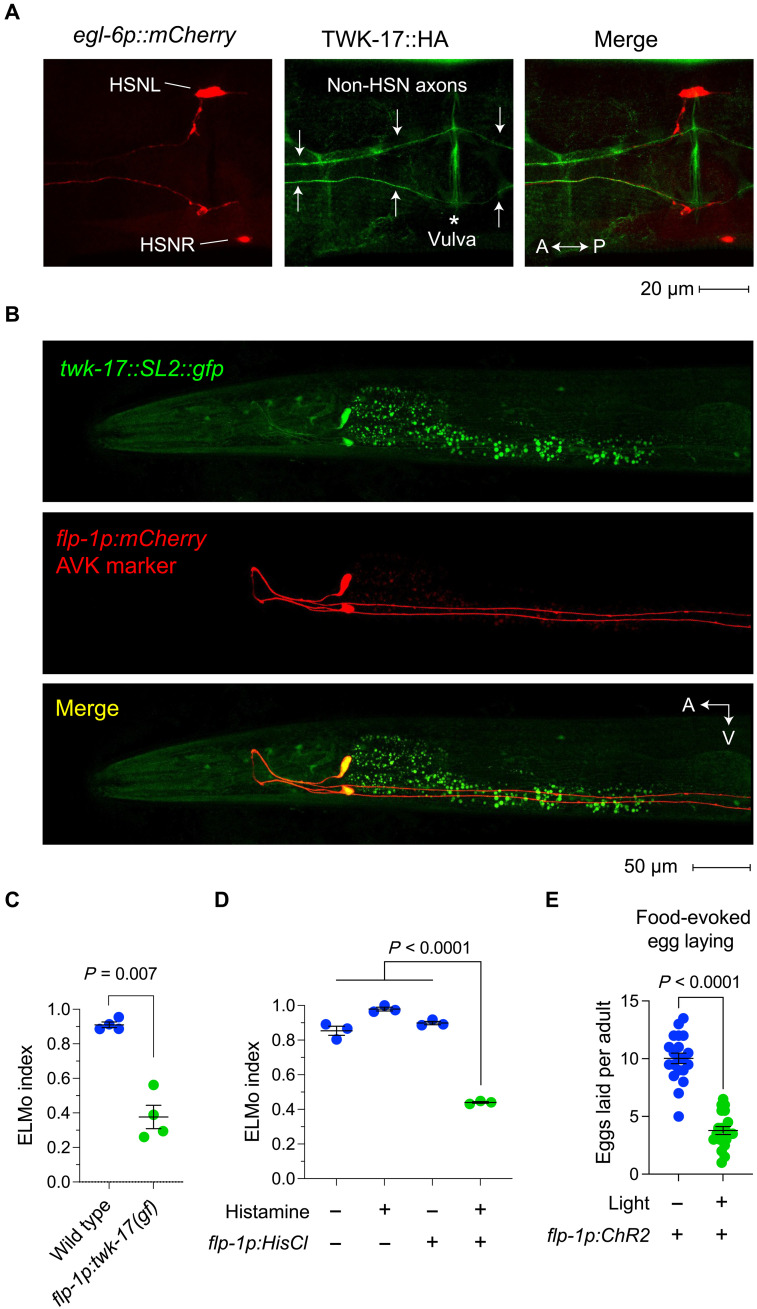
TWK-17(gf) channels silence AVK interneurons, which are critical for modulation of egg laying by food cues. (**A**) Ventral view of an animal immunostained for HA-tagged TWK-17 and mCherry-marked HSNs. Arrows indicate TWK-17–expressing neurites that pass through the egg-laying system. The asterisk indicates the vulva. P, posterior. (**B**) Fluorescence micrograph of an animal carrying a *twk-17::sl2::gfp* reporter locus and an mCherry marker of AVK interneurons. (**C**) ELMo indices of animals expressing *twk-17(gf)* in AVKs using an *flp-1p::twk-17(gf)* transgene and wild-type controls. (**D**) ELMo indices of animals expressing the chemogenetic silencer HisCl1 in AVKs using an *flp-1p::HisCl1* transgene either with or without histamine together with wild-type controls. (**E**) Number of eggs laid on food of *lite-1* mutants expressing channelRhodopsin in AVKs using an *flp-1p::ChR2* transgene either with or without light stimuli. *n* = 20 animals for each condition. In scatterplots, error bars indicate the means ± SEM. *P* values were computed using one-way ANOVA with Tukey’s multiple comparison test.

*twk-17(gf)* mutants fail to suppress egg laying in the absence of food. Because potassium channels like TWK-17 are negative regulators of neuronal excitability, we hypothesized that TWK-17(gf) channels express a circuit that normally provides inhibitory input to the egg-laying neuromusculature. To identify components of this circuit, we used CRISPR/Cas9 gene editing to generate a hemagglutinin (HA)–tagged *twk-17* reporter allele (fig. S3). Following immunostaining with anti-HA antibodies, we observed that TWK-17 was not expressed by cells in the egg-laying system (Fig. 3A). Instead, we observed TWK-17 in neurites that originate in the head and traverse the egg-laying neuromusculature in close apposition to HSN neurites (Fig. 3A). To identify these neurons, we used CRISPR/Cas9 gene editing to generate a transcriptional *twk-17* reporter allele that carries SL2-trans-spliced green fluorescent protein (GFP)–coding sequences in the 3′ untranslated region of the *twk-17* locus (fig. S3). We used this allele to determine the expression of *twk-17* in adults when the neuromusculature of the reproductive system is fully developed. We observed that *twk-17* was prominently expressed in AVK interneurons, although dim fluorescence in other neurons could be seen (Fig. 3B). AVK cell bodies are in the head, and their neurites enter the ventral cord and extend past the egg-laying system (*12*, *35*). To further confirm that *twk-17* is primarily expressed in AVK neurons, we analyzed available mRNA sequencing (mRNAseq) data from AVKs and other neuron types (*22*, *36*–*38*). Differential expression analysis indicated that *twk-17* transcripts are highly enriched in AVKs compared to unsorted neurons, sensory neurons, and monoaminergic neurons (fig. S4). We noted that while our reporter allele indicated that AVKs are a primary site of AVK expression, single-cell RNA sequencing (scRNAseq) data from the CeNGEN project predicted that three neuron types should be as enriched for *twk-17* transcripts as AVKs or more: ALNs, the DB9 motor neurons, and PDBs (*38*). As both small conditional RNA sequencing and the *twk-17::sl2::gfp* allele that we generated report transcription of the endogenous *twk-17* locus, it is not clear why they differ in this regard. It is possible that *twk-17* transcripts are subject to posttranscriptional regulation that does not act on *gfp* transcripts. Both methods, however, indicate that AVKs are highly enriched for *twk-17* transcripts compared to most other neurons, and we therefore focused on these neurons as putative sites of TWK-17 action.

To test whether TWK-17(gf) channels affect egg laying by silencing AVKs, we used the *flp-1* promoter to express the *twk-17(gf)* allele, specifically in AVK neurons (*39*). Expression of TWK-17(gf) in AVKs recapitulated the modulation defect of *twk-17(gf)* mutants (Fig. 3C). To determine whether inhibition of AVKs sufficed to cause defects in modulation of egg laying, we expressed the histamine-gated chloride channel HisCl1 (*40*) to allow chemogenetic silencing of AVKs. Siblings lacking the *HisCl1* transgene or transgenic animals that were not exposed to histamine, which activates HisCl1, had normal egg-laying modulation. By contrast, *HisCl1* transgenics exposed to histamine showed a strong modulation defect (Fig. 3D). We further tested whether AVK activation suffices to inhibit egg laying using optogenetic activation of AVKs in animals exposed to food. We found that optogenetic activation of AVKs using the excitatory opsin channelRhodopsin2 suppressed egg laying when food was available (Fig. 3E). These results indicate that the modulation defect of *twk-17(gf)* mutants results from silencing of AVK interneurons, which transmit inhibitory signals to the egg-laying system.

### Dopaminergic food-sensing neurons inhibit AVKs through a D2-like receptor

**Fig. 4.**
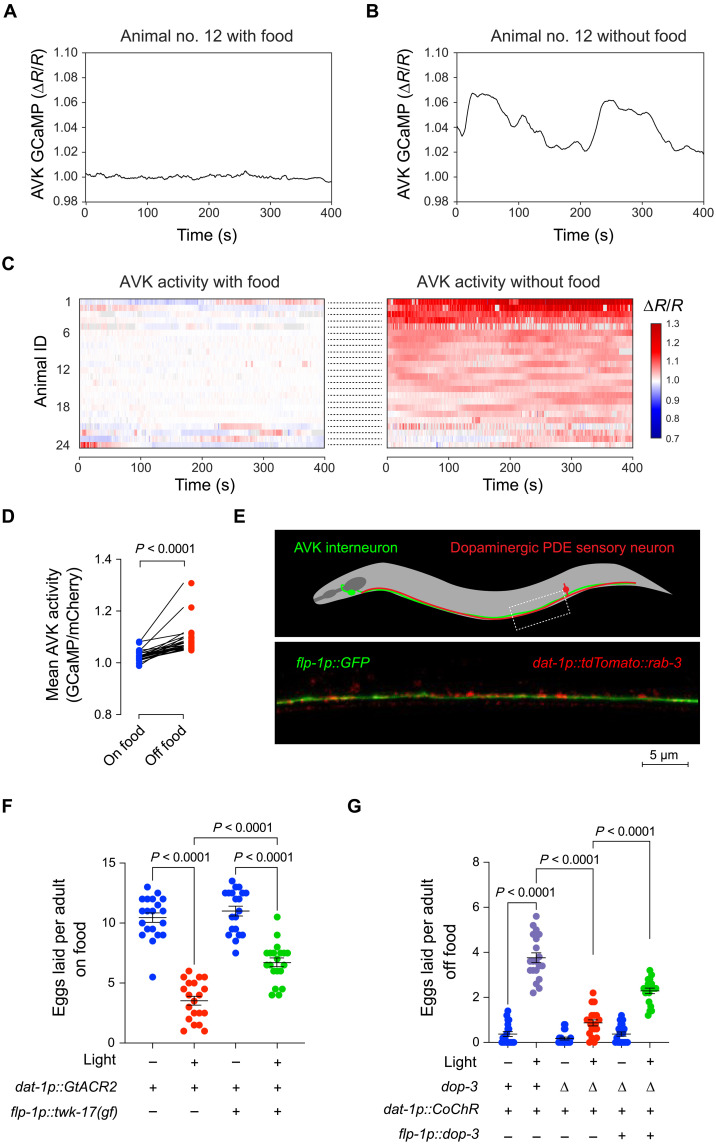
AVKs are inhibited by dopaminergic food-sensing neurons. (**A** and **B**) Representative GCaMP/mCherry fluorescence ratios (Δ*R*/*R*) recorded from AVK neurons in the same animal on food (A) and then off food (B). (**C**) Heatmaps of Δ*R*/*R* traces from AVKs in animals on food and then transferred to off-food conditions. A given row in the two heatmaps shows data from the same animal either on or off food. *n* = 24 animals. (**D**) Mean ∆*R*/*R* values recorded from animals on food and then transferred off food. The *P* value is computed using a paired *t* test. (**E**) Schematic of the organization of AVK and PDE neurites and representative micrograph of tdTomato-tagged RAB-3 puncta in PDE neurons closely apposed to AVK neurites. (**F**) Number of eggs laid in the presence of food by *lite-1* mutants expressing an inhibitory opsin in dopaminergic neurons using *dat-1p::GtACR2* either with or without AVKs silenced by an *flp-1p::twk-17(gf)* transgene. *n* = 20 to 30 animals per condition. (**G**) Number of eggs laid in the absence of food by *lite-1* mutants expressing an excitatory opsin using a *dat-1p::CoChR* transgene either with or without a dop-3 mutation and an *flp-1p::dop-3* transgene that specifically restores *dop-3* expression to AVKs. *n* = 20 to 30 animals per condition. In scatterplots, error bars indicate the means ± SEM. *P* values were computed using one-way ANOVA with Tukey’s multiple comparison test.

Our observations suggested that AVK activity would be affected by detection of food cues. To test this, we measured AVK Ca^2+^ activity in animals foraging in the presence or absence of food. When food was present, AVK calcium was typically stable with few fluctuations (Fig. 4A). By contrast, after transfer to arenas without food, AVK neurons in the same animals displayed elevated Ca^2+^ that often fluctuated over a period of tens of seconds (Fig. 4, B to D, and fig. S5). Recordings from control animals expressing GFP instead of GCaMP in AVKs were not affected by the presence or absence of food (fig. S6). AVK neurons are, therefore, less active in the presence of food stimuli, suggesting that a food-sensing mechanism inhibits AVK neurons to activate egg-laying behavior.

What sensory neurons might detect food to inhibit AVKs? Prior studies and synaptic connectivity within the *C. elegans* nervous system implicated a set of dopaminergic sensory neurons that are directly activated by food: the PDEs. The PDE sensory neurons make extensive synapses onto AVKs (*12*), which is reflected in the colocalization of presynaptic vesicle clusters in PDE neurons and AVK processes in the ventral cord (Fig. 4E). Furthermore, the PDE neurons were shown to modulate locomotion in response to food-sensing by inhibiting AVKs through the D2-like receptor, DOP-3 (*36*, *41*). Last, optogenetic manipulation of dopaminergic neurons was observed to affect *C. elegans* egg-laying behavior (*42*), although the circuit on which dopamine acts was unknown.

To test whether PDE neurons regulate egg-laying behavior via the AVKs, we activated GtACR2, a light-gated anion channel (*43*), expressed in dopaminergic neurons (*42*). Optogenetic inhibition of dopaminergic neurons reduced egg laying under permissive conditions, i.e., when food is present (Fig. 4F). However, when AVKs were silenced by the expression of TWK-17(gf) in AVK neurons, the effect of optogenetic inhibition of dopaminergic neurons was blunted (Fig. 4F). We also measured the effect of optogenetic activation of dopaminergic neurons on egg-laying behavior. Stimulation of dopaminergic neurons with the excitatory opsin CoChR (*42*, *44*) elicited egg laying in the absence of food (Fig. 4G). Stimulating dopaminergic neurons in *dop-3* mutants did not trigger egg laying (Fig. 4G), indicating that D2-like dopamine receptors are critical for dopaminergic modulation of egg laying. Expression of DOP-3 in AVKs restored the effect of dopamine on egg laying to *dop-3* mutants (Fig. 4G). Together, these findings indicate that dopaminergic food-sensing neurons promote egg laying by directly inhibiting the AVKs. We note, however, that we cannot exclude the possibility that other food-sensing mechanisms might provide parallel excitation to the egg-laying system.

### Neuropeptide signals from AVK neurons regulate egg laying

**Fig. 5.**
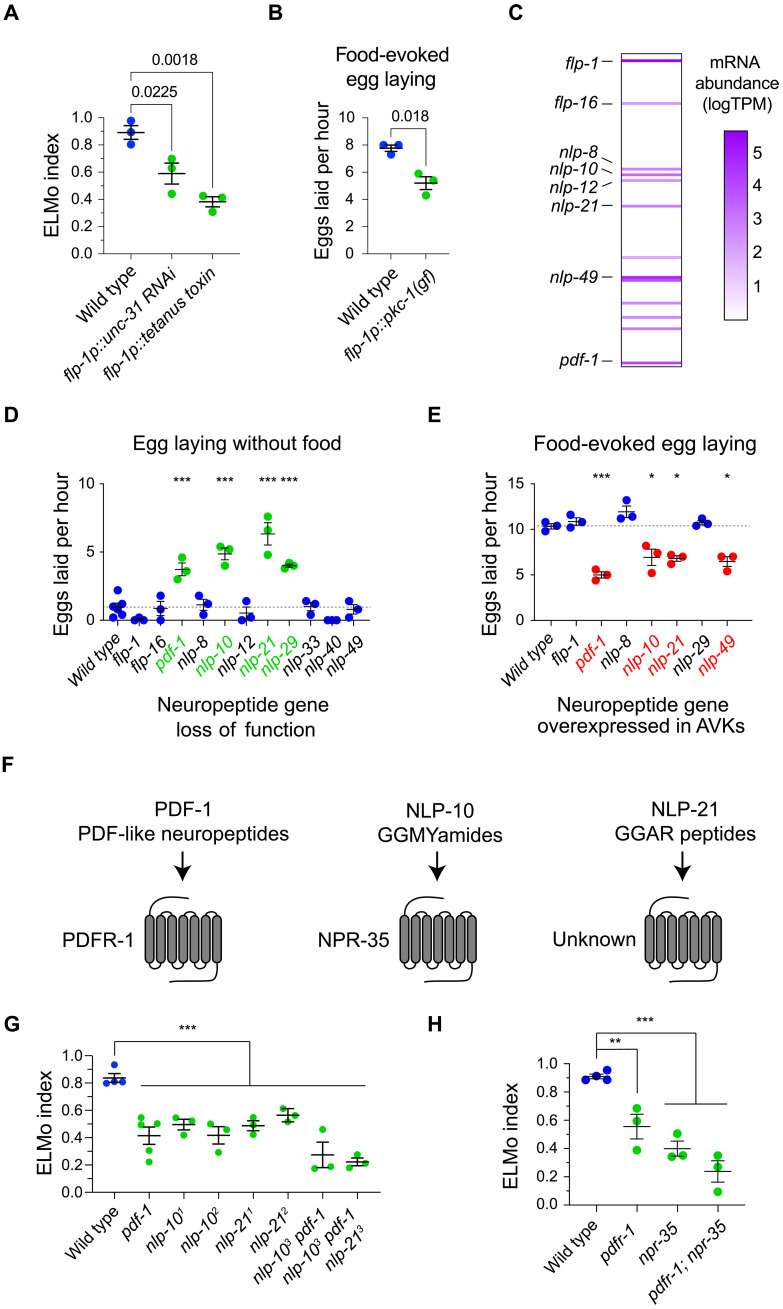
AVKs use a compound neuropeptide signal to regulate egg laying. (**A**) ELMo indices of animals carrying transgenes that specifically disrupt neurosecretion from AVKs and the wild-type controls. (**B**) Number of eggs laid in the presence of food by animals carrying an *flp-1p::pkc-1(gf)* transgene, which potentiates neuropeptide release from AVKs, and the wild-type controls. (**C**) Heatmap plotting neuropeptide gene expression in AVK neurons reported by the CeNGEN project [Taylor *et al.* (*38*)]. (**D**) Number of eggs laid in the absence of food by neuropeptide gene mutants and the wild type. Each point represents one replicate of at least 10 animals. (**E**) Number of eggs laid in the presence of food by animals overexpressing neuropeptides in AVKs using *flp-1p::neuropeptide* transgenes and the wild-type controls. (**F**) Pairings of AVK neuropeptides implicated in the control of egg laying with receptors. (**G**) ELMo indices of single, double, and triple mutants for genes that encode AVK neuropeptides implicated in the control of egg laying. The *nlp-10^1^* allele is *n4544*, *nlp-10^2^* is *tm6232*, *nlp-10^3^* is *wz236*, *nlp-21^1^* is *tm2569*, *nlp-21^2^* is *sy1807*, and *nlp-21^3^* is *wz327*. (**H**) ELMo indices of single and double mutants for receptors for AVK neuropeptides and the wild-type controls. In scatterplots, error bars indicate the means ± SEM. *P* values were computed using one-way ANOVA with Tukey’s multiple comparison test. *P < 0.05; **P < 0.01; ***P < 0.001.

The neurotransmitter identity of AVK neurons remains unknown (*45*, *46*); however, AVK neurons express a large number of neuropeptides and are highly enriched for transcripts encoding factors that mediate neuropeptide synthesis, maturation, and secretion (*36*, *38*, *47*), suggesting that they primarily use neuropeptides to signal to their downstream partners. We tested whether neuropeptide release is required for AVKs to modulate egg laying. First, we blocked neurosecretion from AVKs using the cell-specific expression of the tetanus toxin light chain (*48*). Animals with impaired AVK transmission showed a profound modulation defect (Fig. 5A). We next used AVK-targeted RNAi (RNA interference) to knock down the expression of *unc-31*, which encodes a factor required for the release of transmitters and modulators stored in dense-core vesicles (*49*). Knockdown of *unc-31* in AVKs also caused defects in the modulation of egg laying (Fig. 5A). Last, we tested whether targeted up-regulation of dense-core vesicle fusion in AVKs affected egg-laying behavior. For this, we expressed in AVKs a gain-of-function variant of the protein kinase C isozyme PKC-1, which stimulates dense-core vesicle fusion and neuromodulator release (*50*). AVK-specific expression of PKC-1(gf) inhibited egg laying under permissive conditions, i.e., in the presence of food (Fig. 5B). Together, these data indicate that neuromodulators stored in dense-core secretory vesicles are released by AVK neurons to modulate egg-laying behavior.

Because AVK neurons express many neuropeptides, we hypothesized that peptidergic signaling from AVKs was required to modulate egg-laying behavior. We analyzed AVK transcriptomes acquired from bulk and single-cell mRNAseq (*36*, *38*) to identify candidate neuropeptides. We identified 11 neuropeptide genes, 8 from CeNGEN and 3 from bulk RNAseq, that were expressed at high levels in AVKs and for which there were available mutants (Fig. 5C and fig. S7). We first screened for AVK neuropeptides that modulate egg laying by testing whether neuropeptide mutants failed to inhibit egg release in the absence of food. We found that mutants for four neuropeptide genes—*pdf-1*, *nlp-10, nlp-21,* and *nlp-29*—laid eggs in the absence of food (Fig. 5D). We next screened AVK neuropeptides for the ability to inhibit egg laying under permissive conditions when overexpressed. We found that overexpression in AVKs of *pdf-1*, *nlp-10*, *nlp-21*, and *nlp-49* suppressed egg-laying behavior in the presence of food (Fig. 5E). Three neuropeptide genes—*pdf-1*, *nlp-10*, *and nlp-21*—were identified in both screens of AVK neuropeptide genes. Notably, our screens did not implicate FLP-1 neuropeptides in modulation of egg laying by AVKs even though *flp-1* is the most highly expressed neuropeptide gene in AVKs and FLP-1 peptides are required for AVK regulation of locomotion and foraging (*36*, *41*, *51*, *52*). One neuropeptide gene, *nlp-49*, suppressed egg laying when overexpressed but was not required to prevent egg release in the absence of food. *nlp-49* was previously noted to mediate inhibition of egg-laying behavior (*53*), and our data suggest that NLP-49 peptides might function redundantly with the other AVK neuropeptides identified by our screens.

We focused our subsequent analysis on the three neuropeptide genes that were identified by both screens: *pdf-1*, *nlp-10*, and *nlp-21*. PDF-1 and NLP-10 neuropeptides have been matched to the G protein–coupled receptors PDFR-1 and NPR-35, respectively, and receptors for NLP-21 peptides are not known (Fig. 5F) (*54*, *55*). We measured the effect of mutating these neuropeptide genes singly and in combination. Because *pdf-1*, *nlp-10*, *and nlp-21* are linked, we used CRISPR/Cas9 gene editing to generate strains carrying multiple mutations (fig. S8). As expected, mutants for each neuropeptide gene displayed a modulation defect, but the observed effect of single gene mutations was intermediate to the effects we had observed when silencing AVKs (Fig. 5G). *nlp-10 pdf-1* double mutants displayed a stronger modulation defect than either single mutant (Fig. 5G). Furthermore, introducing an *nlp-21* mutation did not further enhance this modulation defect (Fig. 5G), suggesting that PDF-1 and NLP-10 neuropeptides are more important for AVK modulation of egg laying. Last, we tested whether mutants lacking receptors for PDF-1 and NLP-10 neuropeptides fail to modulate their reproductive behavior in response to food cues. *pdfr-1* and *npr-35* single mutants had intermediate modulation defects, and *pdfr-1; npr-35* double mutants displayed modulation defects that were comparable to those caused by silencing AVKs or mutating multiple AVK neuropeptide genes (Fig. 5H). These data indicate that the neuropeptide signal used by AVKs to modulate egg-laying behavior comprises multiple neuropeptides that act on distinct receptors.

### Neuropeptide signals from AVKs converge on AIY interneurons to modulate egg-laying behavior

To determine what circuits are regulated by AVKs to modulate egg laying, we sought to identify the site of action of the receptors for AVK neuropeptides. The PDF-1 receptor PDFR-1 is widely expressed in the nervous system, including in HSN neurons (*38*, *56*, *57*). Transcripts encoding the receptor for NLP-10 peptides, NPR-35, have also been detected in several neuron types, including VC motor neurons in the egg-laying system (*38*). We used intersectional genetics to (i) identify cells that coexpress these peptide receptors and (ii) test the hypothesis that these receptors function together in cells that coexpress them.

**Fig. 6.**
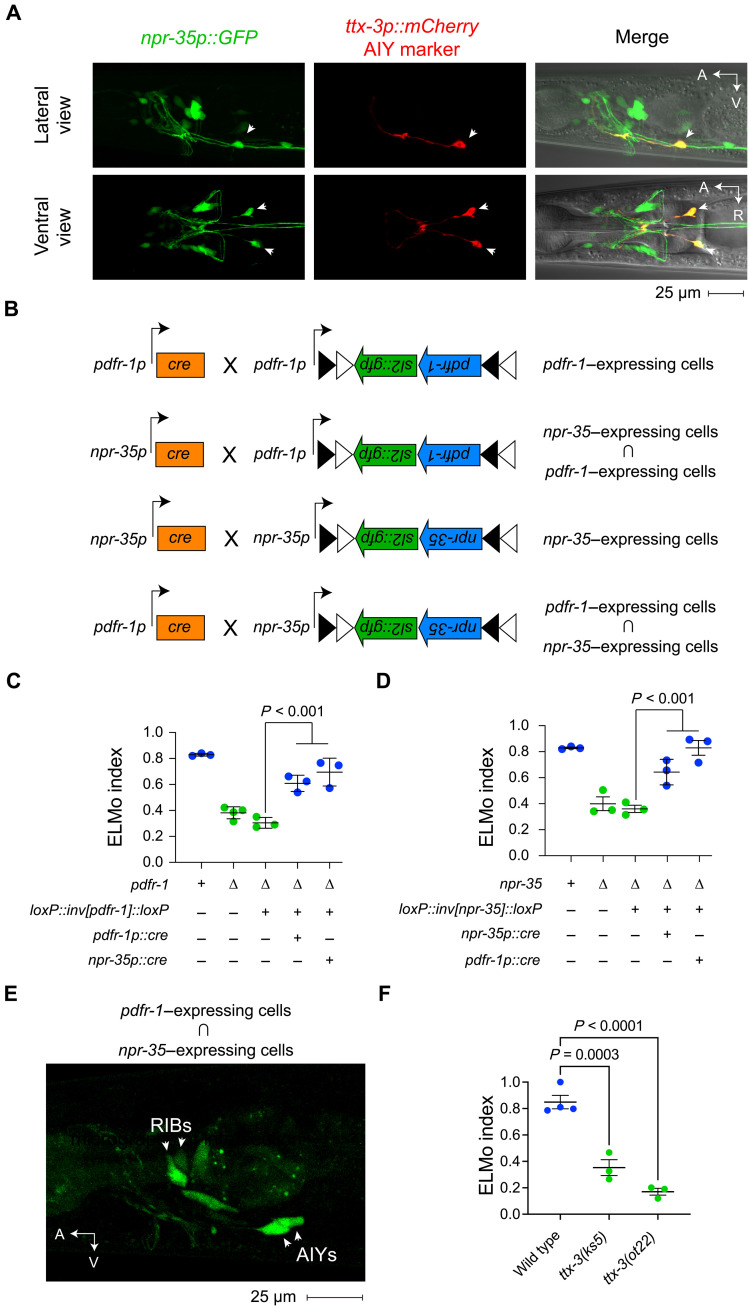
AIY neurons receive neuropeptide signals from AVK to coordinate egg laying and food sensing. (**A**) Micrographs of lateral and ventral views of transgenic animals expressing *npr-35p::gfp* and the AIY marker *ttx-3p::mCherry*. Arrowheads mark AIY neurons. R, right. (**B**) Schematic of transgenes used for intersectional genetics to target neurons coexpressing *pdfr-1* and *npr-35*. (**C**) ELMo indices of *pdfr-1* mutants carrying an inverted *pdfr-1* array with or without a *pdfr-1p::cre* transgene or an *npr-35p::cre* transgene together with *pdfr-1* mutant controls. (**D**) ELMo indices of *npr-35* mutants carrying an inverted *npr-35* array with or without an *npr-35p::cre* transgene or a *pdfr-1p::cre* transgene together with *npr-35* mutant controls. (**E**) Micrographs showing neurons labeled by *npr-35p* and *pdfr-1p* intersectional transgenesis. Arrows and arrowheads indicate AIY and RIB neurons, respectively. (**F**) ELMo indices of the wild type and mutants carrying either the *ks5* missense mutation or the *ot358* nonsense mutation in the AIY specification factor *ttx-3*. Error bars indicate the means ± SEM. *P* values were computed using one-way ANOVA with Tukey’s multiple comparison test.

To begin, we generated an *npr-35p::GFP* transcriptional reporter to mark cells that express NPR-35. We observed prominent expression in head neurons (Fig. 6A). The promoter used for this reporter transgene was active in the egg-laying system but was clearly expressed in neurons known to express *pdfr-1*, for example, the AIY interneurons (Fig. 6A) (*38*). To restore receptor expression to neurons that coexpress PDFR-1 and NPR-35, we generated transgenes that harbor inverted *pdfr-1* or *npr-35* coding sequences in a FLEx cassette and under the control of their respective regulatory sequences (*57*, *58*). We also generated transgenes that use these regulatory sequences to drive the expression of Cre recombinase. In cells expressing Cre, the orientation of the FLEx cassette reverses, and the embedded receptor can be expressed, as depicted in Fig. 6B.

An inverted *pdfr-1* transgene on its own did not restore the modulation defect of *pdfr-1* mutants, but it fully restored modulation of egg laying to *pdfr-1* mutants when combined with a *pdfr-1p::cre* transgene (Fig. 6C). We also observed strong rescue of the *pdfr-1* phenotype when inverted *pdfr-1* was combined with a *rimb-1p::cre* transgene, which drives Cre expression pan-neuronally, indicating that PDFR-1 is required in neurons to modulate egg laying. Notably, inverted *pdfr-1* combined with an *npr-35p::cre* transgene rescued the *pdfr-1* phenotype to the same extent as transgenes that drive Cre expression pan-neuronally (Fig. 6C and fig. S9), indicating that the expression of PDFR-1 is only required in the neurons that coexpress PDFR-1 and NPR-35. We repeated this experiment, this time using a transgene carrying an inverted *npr-35* coding sequence under the control of regulatory sequences from the *npr-35* locus. The inverted *npr-35* transgene on its own did not rescue the modulation defect of *npr-35* mutants, but we observed strong rescue when inverted *npr-35* was combined with an *npr-35p::cre* transgene (Fig. 6D). When we combined inverted *npr-35* with a *pdfr-1p::cre* transgene, we also observed strong rescue of the modulation defect of *npr-35* mutants (Fig. 6D), indicating again that cells coexpressing NPR-35 and PDFR-1 are regulated by AVK neuropeptides.

Cells in which Cre recombinase has rearranged inverted G protein–coupled receptor transgenes were marked by the expression of GFP (Fig. 6B), permitting us to determine the identities of neurons that coexpress PDFR-1 and NPR-35. In animals carrying inverted *npr-35* and *pdfr-1p::cre* transgenes, we observed the expression of GFP only in a small number of head neurons (Fig. 6E) but not in cells of the egg-laying system. AIY neurons were consistently marked by the rearranged *npr-35* transgene, and we observed weaker expression in RIB neurons and variable expression in two to four other head neurons that we did not identify (Fig. 6E). These data suggested that a small set of head neurons was regulated by AVK neuropeptides and sufficed to support modulation of egg laying by food cues. Because they were consistently and strongly marked by the intersectional reporter, we tested whether AIY neurons were necessary for modulation of egg laying. For this, we used *ttx-3* mutants, which are specifically defective in specifying the AIY cell fate during development and lack functional AIY neurons (*59*). Two independently derived *ttx-3* mutations caused modulation defects comparable to those of strains in which neuropeptide signals from AVKs were abolished or the AVK neurons themselves were silenced (Figs. 3C, 5G, and 6F). These data indicate that PDF-1 and NLP-10 neuropeptides released from AVKs converge on AIY interneurons to regulate *C. elegans* reproductive behavior.

## DISCUSSION

**Fig. 7.**
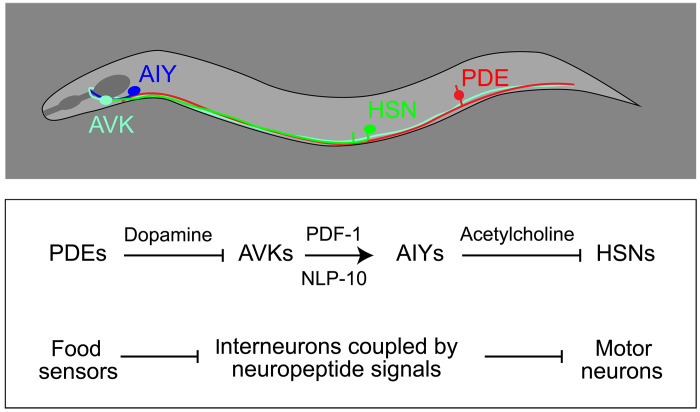
Model of the neuromodulatory circuit that coordinates food sensing and reproductive behavior. Data from this study support a model in which dopaminergic food-sensing neurons make synapses onto and inhibit AVK neurons, which are coupled to AIY neurons by a compound neuropeptide signal. AVK and AIY interneurons in turn inhibit the egg-laying system, possibly via cholinergic signaling.

Our study supports a model in which food-sensing neurons promote reproductive behavior by inhibiting a circuit that constitutively suppresses activity of the egg-laying neuromusculature (Fig. 7). In this model, the inhibitory circuit downstream of dopaminergic food-sensing neurons comprises peptidergic AVK neurons, which receive inputs from food-sensing neurons, and AIY neurons, which express receptors for AVK-derived PDF-1 and NLP-10 neuropeptides. PDF-1–like neuropeptides are well known to mediate excitatory signaling in other contexts (*60*, *61*), consistent with our observation that loss of AVK function and loss of AIY function cause similar modulation defects. Our study further predicts that NLP-10 peptides signaling through the receptor NPR-35 will mediate excitation of AIYs by AVKs. How AIY neurons signal to the neuromusculature of *C. elegans* remains to be determined, but several lines of evidence suggest that AIYs directly inhibit motor neurons in the egg-laying system. First, AIYs make synapses onto HSN motor neurons (*12*), which are critical drivers of egg-laying behavior. Second, AIYs are cholinergic (*62*), and several studies have found that acetylcholine mediates inhibition of the egg-laying system (*9*, *26*). A recent study has found that in response to temperature stimuli, another class of cholinergic interneurons—the AIAs—directly inhibits HSN motor neurons, which express inhibitory cholinergic receptors (*63*). We propose that AIYs function in a similar manner and inhibit HSNs in response to increased AVK activity (Fig. 7).

The circuit that couples food-sensing neurons to the egg-laying system has several features that warrant further consideration. First, the architecture of this modulatory circuit reveals that, by default, the neuromusculature of the reproductive system is inhibited by AVK neurons. Food cues stimulate egg release by inhibiting AVKs and alleviating this default inhibition. In synaptic circuits, this functional architecture is termed “disinhibition” and is found throughout the nervous system. Disinhibition can serve a gating function, as exemplified by the vertebrate basal ganglia and their activation of movement by inhibiting downstream inhibitory circuits (*64*). A key characteristic of disinhibitory circuit motifs is that they contain an additional layer of processing units compared to simpler circuits that use feedforward activation, i.e., A inhibits B, which inhibits C, as opposed to A directly activating C. This extra layer in disinhibitory motifs provides another substrate for regulation and for plasticity mechanisms (*65*). It is especially critical that reproductive behaviors adapt to changing environments and internal states. The extra capacity of disinhibitory networks for regulation might, therefore, explain why the signaling network that modulates *C. elegans* egg laying prominently features disinhibition.

Another remarkable feature of the modulatory circuit revealed by our study is its use of a combination of neuropeptides to relay information from AVK neurons to their downstream partners, the AIYs. AVK neurons have been designated “hub” neurons because they express neuropeptides that act on a large set of neural targets in a manner that is unconstrained by synaptic connectivity (*47*). Previously characterized “spokes” in the AVK hub are specified by one-to-one pairings of neuropeptides and receptors. For example, FLP-1 neuropeptides released from AVKs signal to DMSR-7 receptors in RIM/RIC interneurons to regulate foraging behavior (*52*). AVK neurons also use FLP-1 peptides to control posture by signaling to motor neurons, some of which express the FLP-1 receptor NPR-6 and some of which express the FLP-1 receptor FRPR-7 (*36*, *41*). In the circuit that modulates egg-laying behavior, the AVK-to-AIY connection is specified by the coexpression in AIYs of two receptors—PDFR-1 and NPR-35—for two distinct AVK neuropeptides—PDF-1 peptides and NLP-10 peptides. Functional connections in neuropeptide signaling networks can, therefore, be determined by combinations of neuropeptides and receptors, which vastly increases the number of possible connections these networks can generate.

Last, our study highlights a molecular mechanism that tunes neuromodulation in the circuit that controls *C. elegans* egg laying and is found in neural circuits in animals from many phyla. We found that the excitability of AVK interneurons is likely regulated by the K_2P_-family potassium channel TWK-17. K_2P_ channels were originally identified as low-conductance “leak” channels that can modify neuronal resting potential and excitability, but they have emerged as specific effectors of second messenger signaling pathways and as mediators of neuromodulation (*66*). Seminal studies of *Aplysia* circuits showed that serotonin and neuropeptides exert their effects on synaptic sensitization by regulating potassium channels that have definitive attributes of K_2P_ channels (*67*, *68*). In vertebrates, G protein–coupled receptors for neurotransmitters and neuromodulators regulate K_2P_ channels via canonical second messenger signals, e.g., phosphoinositides and cyclic adenosine 5′-monophosphate (*66*). In *Drosophila*, K_2P_ channels are critical regulators of dopaminergic circuits that control sleep (*69*), and in *C. elegans*, K_2P_ channels have been implicated in the control of γ-aminobutyric acid and dopamine signaling (*22*, *70*). K_2P_ channels can also be regulated by metabolic and physiological signals and have been suggested to function as interoceptors that couple circuit activity to an animal’s internal state (*71*). Food-seeking behaviors are markedly changed by population density and metabolic state (*20*, *72*), and it is likely that reproductive behaviors and how they are controlled are subject to similar context-dependent tuning. An intriguing possibility is that TWK-17 channels in AVK interneurons are regulated either by neuromodulatory input from other circuits or by an interoceptive signal to determine how tightly food-sensing and reproductive behaviors are coupled.

In summary, our study has revealed the functional architecture and molecular components of a neuromodulatory system that controls *C. elegans* reproductive behavior. We suggest that the neurochemical logic of this system, which uses disinhibition and combinatorial neuropeptide signals to relay information about food availability to the motor circuit that controls reproductive behavior, illustrates general principles of neuromodulation. Determining how these principles are instantiated in molecular processes will advance the understanding of neurological and psychiatric disorders rooted in dysfunction of neuromodulatory systems and generate new opportunities to develop therapeutic interventions that target neuromodulation in vivo.

## MATERIALS AND METHODS

*C. elegans* was maintained on nematode growth medium (NGM) plates seeded with *Escherichia coli* OP50 bacteria at 20°C, as described previously (*73*). Mutant alleles and transgenic strains used in this study are listed in table S1.

### Whole-genome sequencing and variant discovery

Genomic DNA was isolated from *egl-6(n592) twk-17(n5210)* mutants and sequenced as previously described (*28*). Mutations were identified using the Genome Analysis Tool Kit (*74*) and analyzed for impact using SnpEff (*75*).

### Plasmid generation

Plasmids for *C. elegans* transgenesis were generated using Gibson cloning. Plasmids and primers used in this study are listed in tables S1 and S2. Primers were purchased from Integrated DNA Technologies, IA. *twk-17a* cDNA was cloned from N2 cDNA synthesized from total RNA isolated using the RNeasy Mini Kit (74104, Qiagen) and reverse transcribed using the Superscript III First Strand Synthesis System for reverse transcription polymerase chain reaction (18080051, Thermo Fisher Scientific). The *twk-17a(G401V)* mutation was introduced using site-directed mutagenesis. *flp-1*, *twk-17*, *pdfr-1*, and *npr-35* 5′ regulatory sequences and neuropeptide genomic sequences were obtained from WormBase (*76*) and polymerase chain reaction amplified from N2 genomic DNA.

### Generation of transgenic and mutant *C. elegans* strains

Transgenic animals were generated by microinjection as previously described (*77*). A complete list of injected constructs and their concentrations can be found in table S3. AVK-specific transgenes were generated by fusing effector proteins to the *flp-1* promoter and coinjected with *flp-1p::GFP* or *mCherry*, as previously described (*39*). At least three independent transgenic lines were tested. *twk-17(wz206)*, *twk-17(wz207)*, *twk-17(wz212)*, *twk-17(wz213)*, *lite-1(wz245)*, *nlp-10(wz236)*, and *nlp-21(wz237)* alleles were generated by CRISPR/Cas9–mediated mutagenesis using a *dpy-10(gf)* co-CRISPR strategy (*78*), and edits were confirmed by Sanger sequencing. *twk-17(wz206)*, *twk-17(wz207)*, and *lite-1(wz245)* were generated by inserting a *STOP-IN* cassette to generate loss-of-function alleles (*32*). HA tag coding sequences were inserted into the 3′ end of the *twk-17* coding sequence to generate *twk-17(wz212)*. An *SL2::GFP* cassette was amplified from the vector pSM (provided by C. Bargmann, Rockefeller University) and inserted to the 3′ untranslated region of the *twk-17* locus to generate *twk-17(wz213)*. *nlp-10(wz236)* and *nlp-21(wz237)* were generated by removing the first coding exon of their respective loci. All CRISPR transgenics were outcrossed to wild-type animals at least three times before testing. All CRISPR reagents were obtained from Integrated DNA Technologies, IA.

### Functional imaging of *C. elegans* neurons

For HSN imaging, young adult transgenics were mounted on 5% agarose pads made in M9 buffer (22 mM KH_2_PO_4_, 22 mM Na_2_HPO_4_, 85 mM NaCl, and 1 mM MgSO_4_), immobilized by focal application of Surgi-Lock veterinary surgical glue (Meridian Animal Health, NE), and placed under a coverslip. For AVK imaging, animals expressing both mCherry and GCaMP6f (or GFP as control) in AVKs were placed on a 2% NGM agarose pad with or without food, and a coverglass was added on top of a Gene Frame (AB0578, Thermo Fisher Scientific) to create a chamber with dimensions of 17 by 28 by 0.25 mm. Animals were tracked with bacterial food and then transferred to plain NGM plates for 2 hours before being tracked again under no-food condition. Videos were taken using a 10× air objective [numerical aperture (NA), 0.3] on a Zeiss Axio Imager M2 (Zeiss, Germany). mCherry and GCaMP6f were excited for 20 ms by alternating 550- and 470-nm light, respectively, using a light-emitting diode light engine (pE-800; CoolLED, UK), and images were acquired with a cooled charge-coupled device camera (Andor; Oxford Instruments, UK). Images were acquired at 3.5 frames per second for 6 min. The microscope, camera, and excitation light source were controlled by μManager. Fluorescence signals were segmented using the ilastik pixel classifier (*79*) after training with more than 300 frames. Regions of interest with a 13-pixel radius centered on the AVK cell body were generated and analyzed in FIJI (*80*). For each animal, the green-to-red fluorescence ratios (*R*) over time were calculated and normalized to the mean ratio measured from the on-food condition.

### Measurements of egg-laying behavior and its modulation

Developmental stages of newly laid eggs were determined as previously described (*28*). For video tracking of egg-laying behavior, young adults were placed on NGM plated with a thin layer of bacterial food. Videos were taken at one frame per second for 2 hours, and eggs were manually identified. To measure the modulation of egg-laying behavior, 1-day-old hermaphrodites were placed singly either on an unseeded NGM plate or on an NGM plate with a thin lawn of *E. coli* OP50. Animals were allowed to lay eggs for 2 hours at room temperature and then removed. Eggs were counted, and for each cohort, an ELMo index was calculated as follows: $\text{ELMo index}=\frac{\text{Eggs laid}\;\text{on}\;\text{food}-\text{Eggs laid}\;\text{off}\;\text{food}}{\text{Eggs laid}\;\text{on}\;\text{food}+\text{Eggs laid}\;\text{off}\;\text{food}}$. Twenty animals were used for each trial (10 on food and 10 off food), and data were collected from at least three independent trials performed on different days. All trials were performed with paired wild-type controls.

### Immunochemistry

Worms were fixed with formaldehyde and permeabilized in two independent batches per genotype using the freeze-cracking method, followed by immunostaining as previously described (*81*). Monoclonal rabbit anti-HA antibodies (C29F4, Cell Signaling Technology) were used at a 1:300 dilution, followed with goat anti-rabbit secondary antibodies conjugated to Alexa Fluor 488 (A11070, Thermo Fisher Scientific) at a 1:500 dilution. Immunostained worms were mounted in Vectashield (H-1200, Vector Laboratories) for imaging. Fluorescence and differential interference contrast micrographs were captured using a Zeiss LSM700 laser-scanning confocal microscope with a 40× oil objective (NA, 1.4). The microscope, camera, and excitation light source were controlled via ZEN software (Zeiss, Germany). Confocal image stacks were collected with 0.5-μm *Z*-steps.

### Optogenetic activation or silencing of neurons

Transgenic *lite-1(ce314)* mutants (*82*) expressing ChR2, CoChR, or GtACR2 were cultivated on NGM plates containing 100 μM all-trans-retinal (R2500, Sigma-Aldrich) for at least two generations. One-day-old adults were used for egg-laying assays described above. During behavior assays, worms were continuously illuminated with 490-nm light (M490L4, Thorlabs) at an irradiance of 5 mW/mm^2^ for 2 hours, after which released eggs were counted.

### Chemogenetic silencing with HisCl transgenes

NGM plates containing histamine (H7250, Sigma-Aldrich) were prepared as previously described (*40*). One-day-old transgenic animals or their nontransgenic siblings were placed on NGM plates or NGM plates containing 10 mM histamine with or without an *E. coli* OP50 lawn for 2 hours at room temperature. Behavior assays were scored as described above.

### Analysis of mRNAseq data to identify AVK neuropeptides

The AVK transcriptome (*36*) and transcriptomes of dopaminergic neurons, serotonin neurons, and BAG neurons (*22*, *37*) were previously reported. Read coverage histograms were generated from genomic alignments using Integrative Genomics Viewer (*83*). AVK-enriched neuropeptide–encoding transcripts were identified using DESeq2 (*84*).

### Microscopy

Young adults were anesthetized with 150 mM NaN_3_ and mounted on 2% agarose pads made in M9 buffer (22 mM KH_2_PO_4_, 22 mM Na_2_HPO_4_, 85 mM NaCl, and 1 mM MgSO_4_). *Z*-Stacks were obtained with a Zeiss LSM800 confocal microscope (Zeiss, Germany) configured with a 40× oil objective (NA, 1.4), and maximum-projection images were created using FIJI (*80*). The microscope, camera, and excitation light source were controlled by ZEN (Zeiss, Germany).

### Statistical analysis

For developmental stages of newly laid eggs, a nonparametric Wilcoxon Mann-Whitney rank sum test was used. ELMo indices and other egg-laying data were analyzed by unpaired Student’s *t* test or ordinary one-way analysis of variance (ANOVA) with Tukey’s multiple comparison test. AVK calcium imaging data were analyzed by paired Student’s *t* test. A complete list of statistical comparisons can be found in table S4. Statistical analyses of all data were performed using GraphPad Prism 10 (GraphPad Software, CA).

## Supplementary Materials

### The PDF file includes:

Figs. S1 to S9 Legends for tables S1 to S4 References
