# Supplementary material for: Food sensing controls C. elegans reproductive behavior by neuromodulatory disinhibition
Source: Sci Adv. 2025 Apr 16;11(16):eadu5829. doi: 10.1126/sciadv.adu5829 (PMC12002139; doi:10.1126/sciadv.adu5829)
Supplement: Supplementary file 1 — Figs. S1 to S9 Legends for tables S1 to S4 References [file sciadv.adu5829_sm.pdf]

Science Advances
AAAS

Supplementary Materials for

Food sensing controls *C. elegans* reproductive behavior by neuromodulatory disinhibition

Yen-Chih Chen *et al.*

Corresponding author: Niels Ringstad, niels.ringstad@med.nyu.edu

Sci. Adv. **11**, eadu5829 (2025)
DOI: 10.1126/sciadv.adu5829

The PDF file includes:

Figs. S1 to S9
Legends for tables S1 to S4
References

Other Supplementary Material for this manuscript includes the following:

Tables S1 to S4

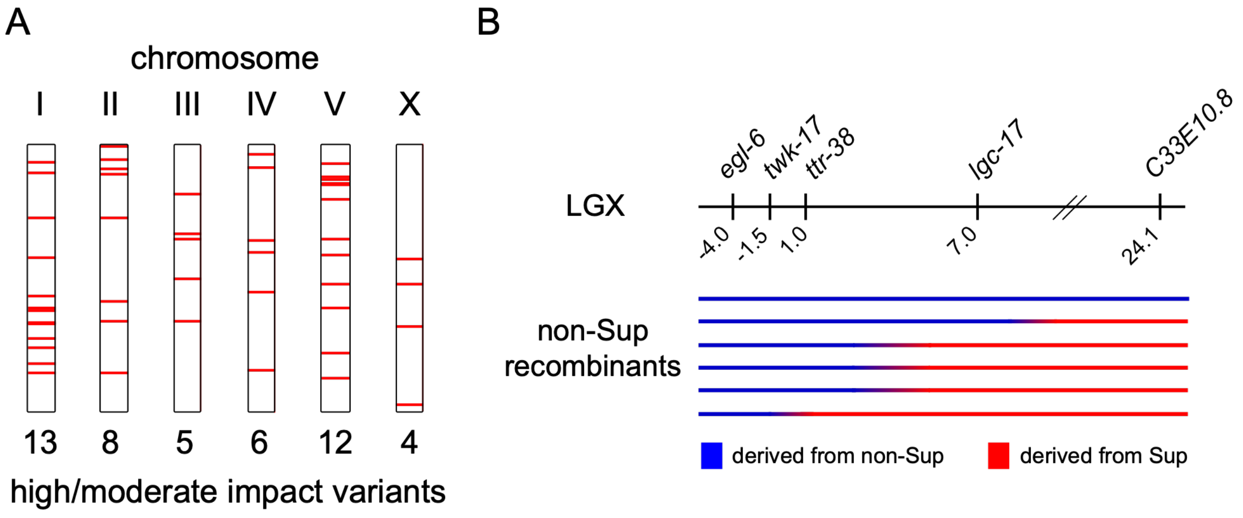

Supplementary Figure 1. The *n5210* mutation affects the K_{2P} gene *twk-17*.

(A) Positions of mutations predicted to affect protein-coding sequences in *egl-6(gf) sup(n5210)* animals are denoted with red hash marks. Their numbers are denoted under each linkage group.

(B) *twk-17*, *ttr-38*, *lgc-17,* and *C33E10.8* are predicted to affect protein-coding on linkage group X. Sanger sequencing at each locus of the six non-Sup progeny showed that they all carried wild-type derived sequence only at *twk-17* locus. Blue = non-Sup derived allele; Red = Sup-derived allele.

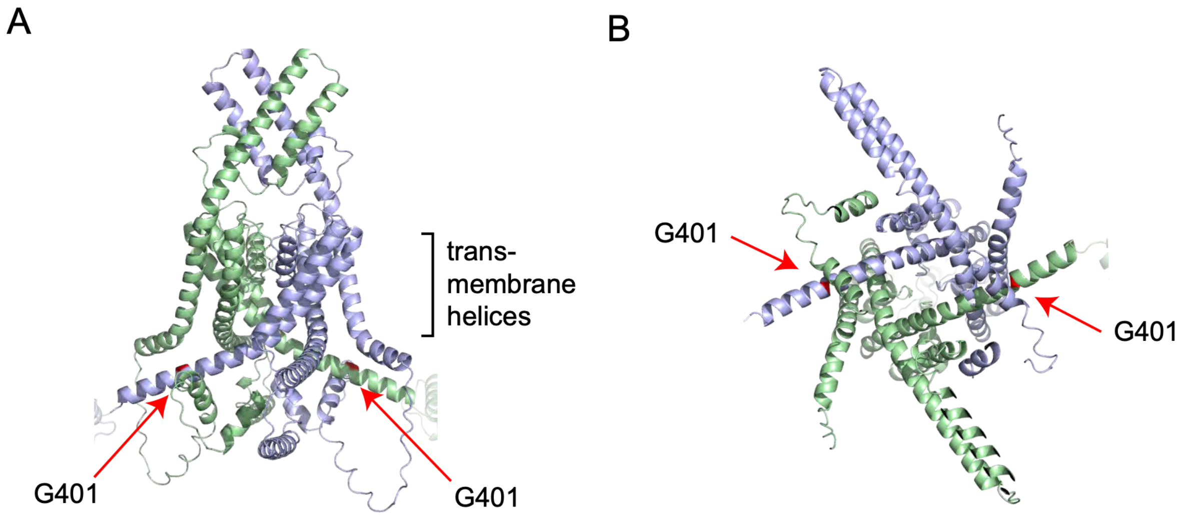

Supplementary Figure 2. *n5210* affects residues in a predicted gate of the K_{2P} channel TWK-17.

(A-B) Lateral and *en face* views of a TWK-17 structure predicted by AlphaFold (85). The glycine residue affected by the *n5210* mutation is predicted to be near the X-gate motif, a motif that controls the opening and closing of the channel pore.

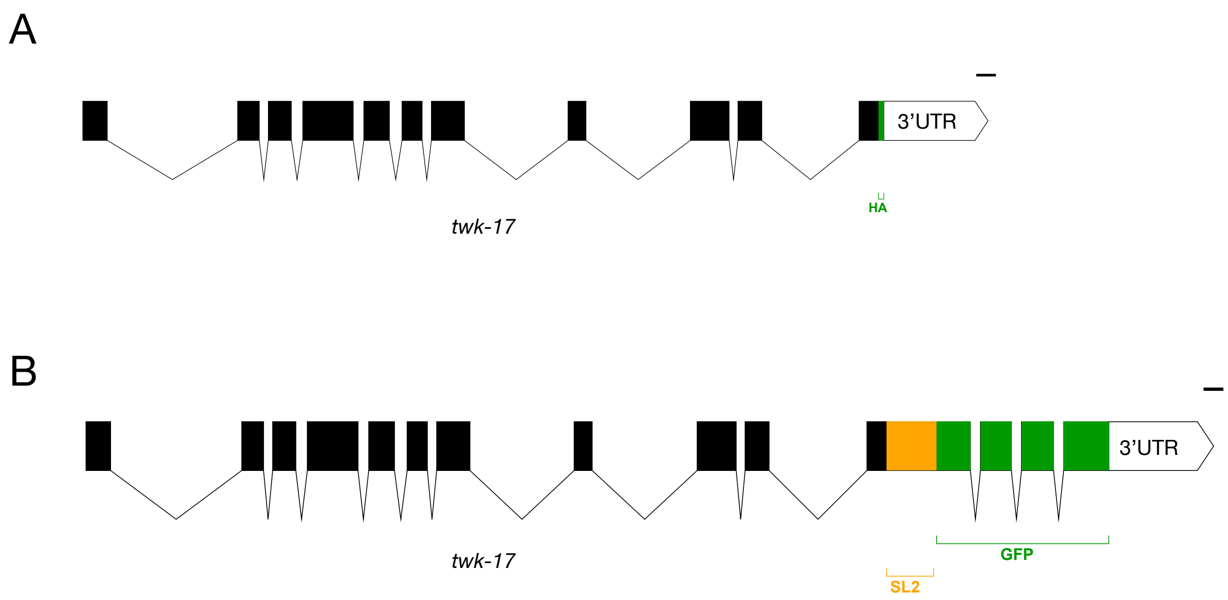

Supplementary Figure 3. Schematics of *twk-17::HA* and *twk-17::SL2::GFP* alleles for identifying endogenous *twk-17* expression.

(A-B) Schematic of *twk-17::HA* (A) and *twk-17::sl2::gfp* (B) alleles. Both alleles were generated using CRISPR/Cas9 genome editing to insert an HA tag or an SL2:: GFP cassette for bicistronic expression of *twk-17* and *gfp* into the endogenous *twk-17* locus. Scale bar indicates 100 bp.

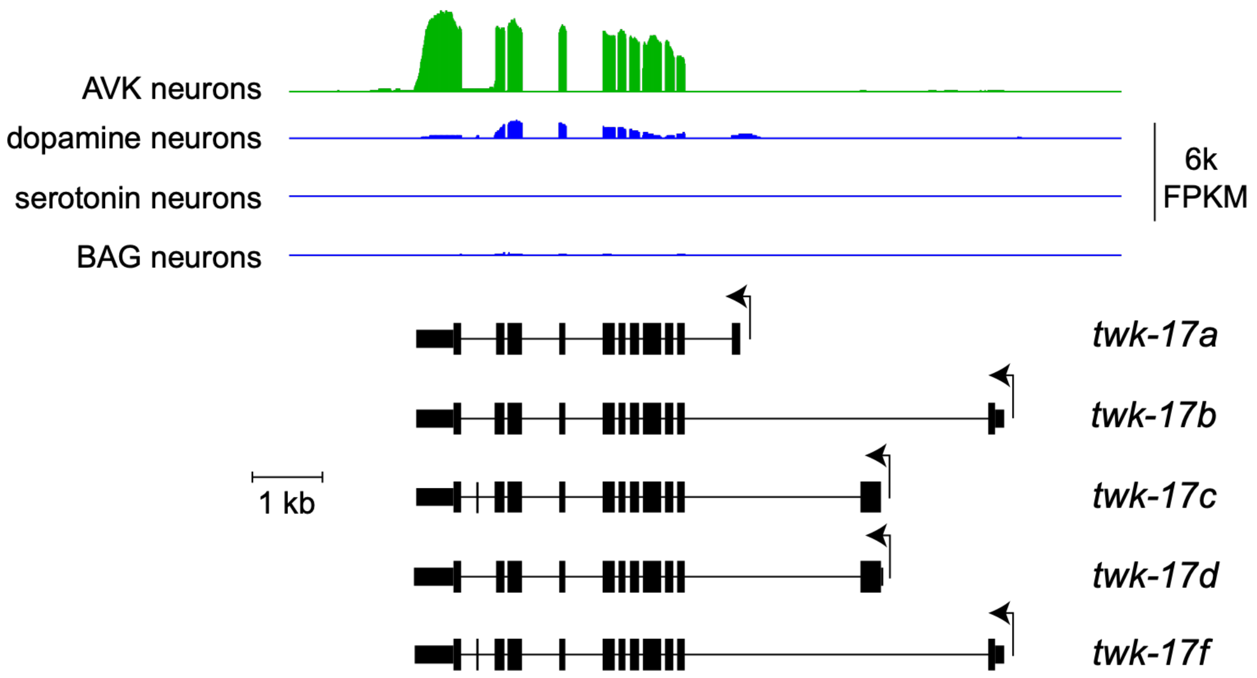

Supplementary Figure 4. *twk-17* is enriched in AVK interneurons. Expression of *twk-17* transcripts in AVK, serotonin, dopamine, or BAG neuron-enriched total RNA sequencing. Read counts are normalized and plotted as fragments per kilobase of transcript per million mapped reads (FPKM).

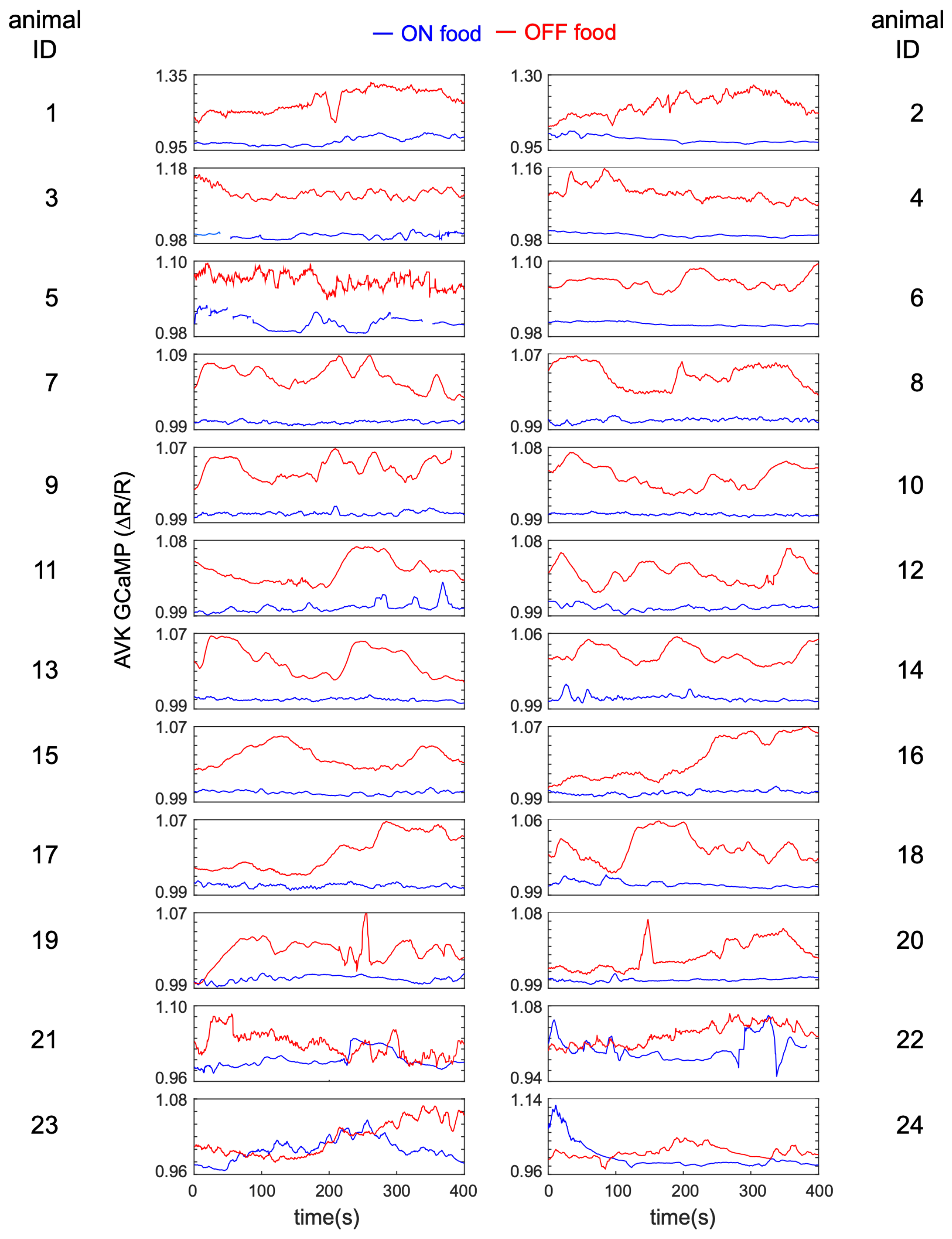

Supplementary Figure 5. Activity of AVK neurons is regulated by food cues.
Traces of GCaMP signals were measured from AVK neurons in 24 individuals in the presence of food (blue) and after they had been removed from food (red).

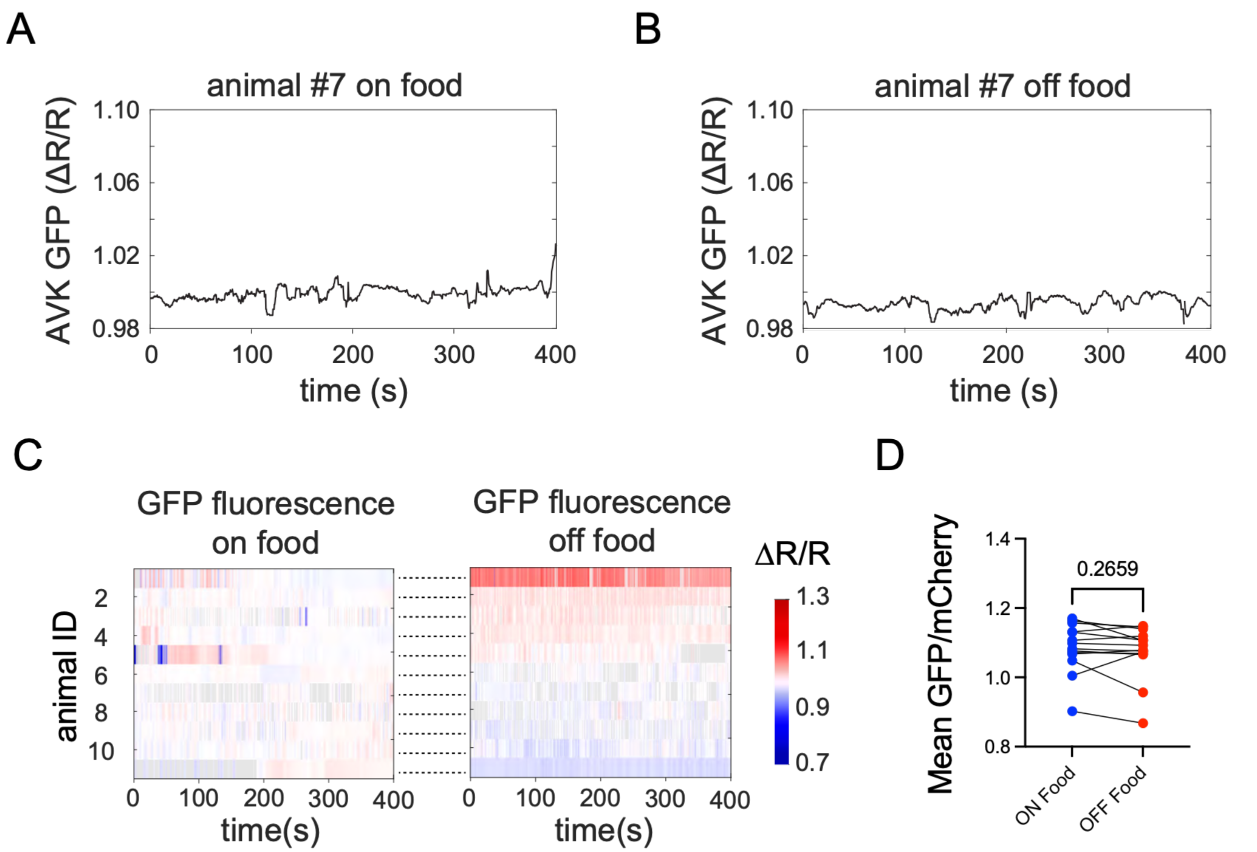

Supplementary Figure 6. GFP signals in AVK interneurons are not altered by food cues. (A-B) Representative ΔR/R traces of GFP fluorescence in AVK neurons from animals on food (A) and animals off food (B).

(C-D) Heatmap of paired ΔR/R traces (C) and mean ΔR/R (D) of GFP fluorescence in AVK from animals on food or off food conditions. N = 11 animals. The P value was computed using a paired *t*-test.

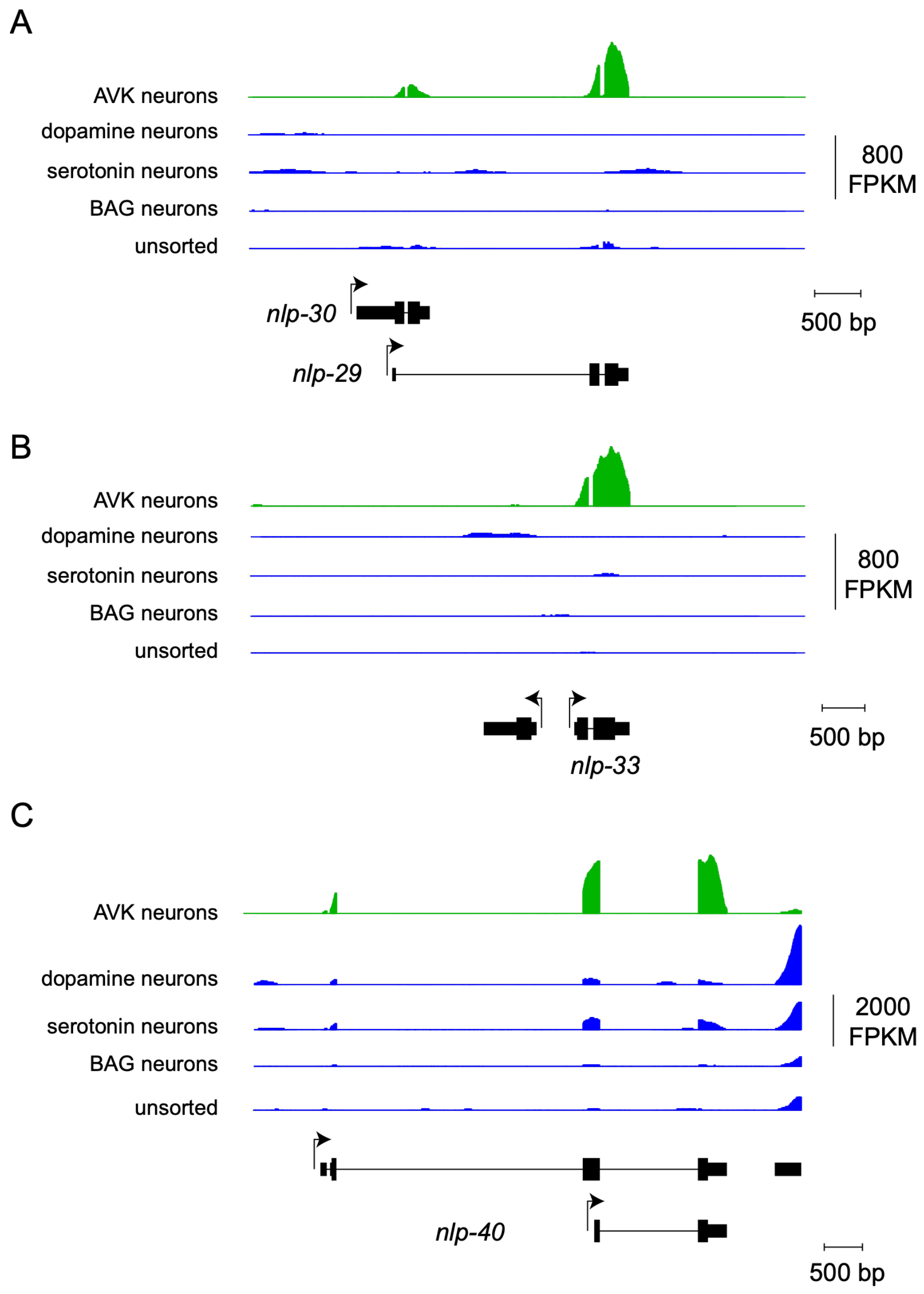

Supplementary Figure 7. Bulk mRNAseq transcriptomes identify three AVK-enriched neuropeptide transcripts that were not revealed by scRNAseq data.
(A-C) Expression of *nlp-29* (A), *nlp-33* (B), and *nlp-40* (C) transcripts in AVK, serotonin, dopamine, or BAG neuron-enriched or unsorted total RNA sequencing. Read counts are normalized and plotted as fragments per kilobase of transcript per million mapped reads (FPKM).

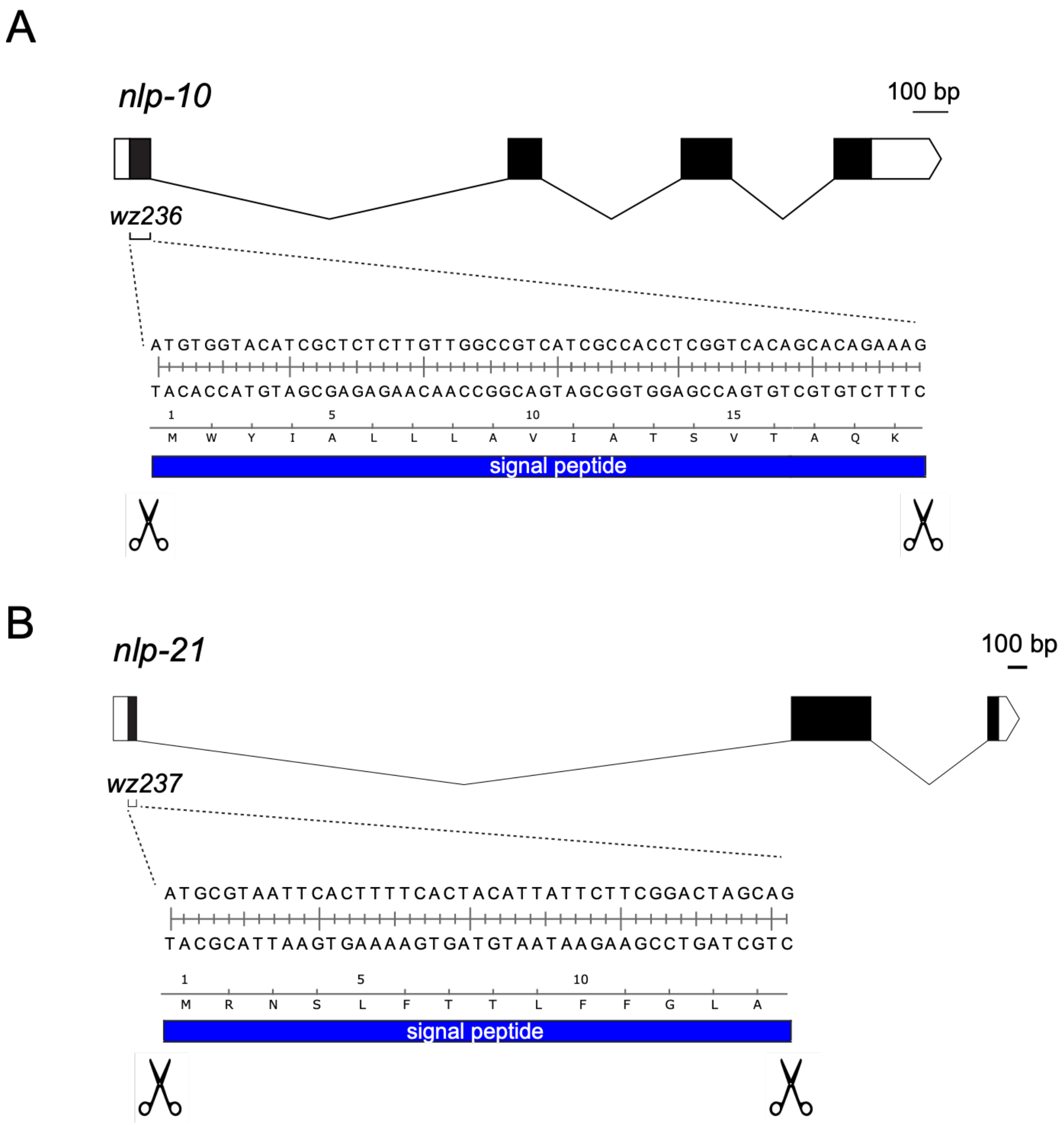

Supplementary Figure 8. Schematics of *nlp-10* and *nlp-21* deletion alleles used to make mutants lacking multiple AVK neuropeptides.

(A-B) Schematic of *nlp-10(wz236)* (A) and *nlp-21(wz237)* (B) alleles. Both alleles were generated by removing the first exon, including the start codon and signal peptide, and creating a frameshift by CRISPR editing.

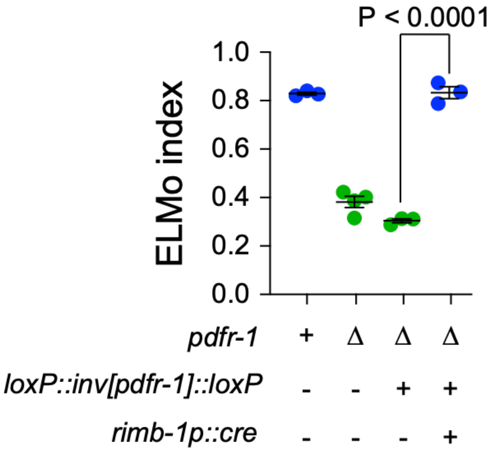

Supplementary Figure 9. Expression of *pdfr-1* in neurons rescued the modulation defect caused by *pdfr-1* mutation.

ELMo indices *pdfr-1* mutants carrying an inverted *pdfr-1* array either with or without the *rimb1p::cre* transgene, which drives expression of Cre recombinase specifically in neurons. Each point represents one replicate of 10 animals on food and 10 animals off food. P values were computed using a one-way ANOVA with Tukey's multiple comparison test. Error bars indicate the mean ± standard error of the mean. ***, $P < 0.001$.

Supplementary Table S1

List of reagents, strains, and plasmids used in this study

Supplementary Table S2

List of primers and CRISPR guide sequences used in this study

Supplementary Table S3

Concentrations of plasmids used to generate transgenic animals

Supplementary Table S4

P values computed from comparisons of data in each figure panel.

REFERENCES AND NOTES

1. F. Grieco, A. J. van Noordwijk, M. E. Visser, Evidence for the effect of learning on timing of reproduction in blue tits. *Science* **296**, 136–138 (2002).

2. R. Spence, G. Gerlach, C. Lawrence, C. Smith, The behaviour and ecology of the zebrafish, Danio rerio. *Biol. Rev. Camb. Philos. Soc.* **83**, 13–34 (2008).

3. C.-H. Yang, P. Belawat, E. Hafen, L. Y. Jan, Y.-N. Jan, Drosophila egg-laying site selection as a system to study simple decision-making processes. *Science* **319**, 1679–1683 (2008).

4. H. R. Horvitz, M. Chalfie, C. Trent, J. E. Sulston, P. D. Evans, Serotonin and octopamine in the nematode Caenorhabditis elegans. *Science* **216**, 1012–1014 (1982).

5. C. Trent, N. Tsung, H. R. Horvitz, Egg-laying defective mutants of the nematode *Caenorhabditis elegans*. *Genetics* **104**, 619–647 (1983).

6. J. Chen, E. P. Caswell-Chen, Facultative vivipary is a life-history trait in Caenorhabditis elegans. *J. Nematol.* **36**, 107–113 (2004).

7. W. R. Schafer, “Egg-laying,” in *WormBook* (The *C. elegans* Research Community, 2005),pp. 1–7.

8. L. E. Waggoner, G. T. Zhou, R. W. Schafer, W. R. Schafer, Control of alternative behavioral states by serotonin in Caenorhabditis elegans. *Neuron* **21**, 203–214 (1998).

9. I. A. Bany, M.-Q. Dong, M. R. Koelle, Genetic and cellular basis for acetylcholine inhibition of Caenorhabditis elegans egg-laying behavior. *J. Neurosci.* **23**, 8060–8069 (2003).

10. M. Zhang, S. H. Chung, C. Fang-Yen, C. Craig, R. A. Kerr, H. Suzuki, A. D. Samuel, E. Mazur, W. R. Schafer, A self-regulating feed-forward circuit controlling C. elegans egg-laying behavior. *Curr. Biol.* **18**, 1445–1455 (2008).

11. N. Banerjee, R. Bhattacharya, M. Gorczyca, K. M. Collins, M. M. Francis, Local neuropeptide signaling modulates serotonergic transmission to shape the temporal organization ofC. elegans egg-laying behavior. *PLOS Genet.* **13**, e1006697 (2017).

12. J. G. White, E. Southgate, J. N. Thomson, S. Brenner, The structure of the nervous system of the nematode Caenorhabditis elegans. *Philos. Trans. R. Soc. Lond. B Biol. Sci.* **314**, 1–340 (1986).

13. Y.-C. Huang, J. Luo, W. Huang, C. M. Baker, M. A. Gomes, B. Meng, A. B. Byrne, S. W. Flavell, A single neuron in C. elegans orchestrates multiple motor outputs through parallel modes of transmission. *Curr. Biol.* **33**, 4430–4445.e6 (2023).

14. M. J. Alkema, M. Hunter-Ensor, N. Ringstad, H. R. Horvitz, Tyramine functions independently of octopamine in the Caenorhabditis elegans nervous system. *Neuron* **46**, 247–260 (2005).

15. A. M. Jose, I. A. Bany, D. L. Chase, M. R. Koelle, A specific subset of transient receptor potential vanilloid-type channel subunits in Caenorhabditis elegans endocrine cells function as mixed heteromers to promote neurotransmitter release. *Genetics* **175**, 93–105 (2007).

16. K. M. Collins, A. Bode, R. W. Fernandez, J. E. Tanis, J. C. Brewer, M. S. Creamer, M. R. Koelle, Activity of the *C. elegans* egg-laying behavior circuit is controlled by competing activation and feedback inhibition. *eLife* **5**, e21126 (2016).

17. J. C. Brewer, A. C. Olson, K. M. Collins, M. R. Koelle, Serotonin and neuropeptides are both released by the HSN command neuron to initiate Caenorhabditis elegans egg laying. *PLOS Genet.* **15**, e1007896 (2019).

18. A. Lin, S. Qin, H. Casademunt, M. Wu, W. Hung, G. Cain, N. Z. Tan, R. Valenzuela, L. Lesanpezeshki, V. Venkatachalam, C. Pehlevan, M. Zhen, A. D. T. Samuel, Functional imaging and quantification of multineuronal olfactory responses in C. elegans. *Sci. Adv.* **9**, eade1249 (2023).

19. B. Brissette, L. Ficaro, C. Li, D. R. Jones, S. Ramanathan, N. Ringstad, Chemosensory detection of polyamine metabolites guides *C. elegans* to nutritive microbes. *Sci. Adv.* **10**, eadj4387 (2024).

20. E. R. Sawin, R. Ranganathan, H. R. Horvitz, C. elegans locomotory rate is modulated by the environment through a dopaminergic pathway and by experience through a serotonergic pathway. *Neuron* **26**, 619–631 (2000).

21. B. Han, Y. Dong, L. Zhang, Y. Liu, I. Rabinowitch, J. Bai, Dopamine signaling tunes spatial pattern selectivity in C. elegans. *eLife* **6**, e22896 (2017).

22. A. Fok, B. Brissette, T. Hallacy, H. Ahamed, E. Ho, S. Ramanathan, N. Ringstad, High-fidelity encoding of mechanostimuli by tactile food-sensing neurons requires an ensemble of ion channels. *Cell Rep.* **42**, 112452 (2023).

23. J. L. Rhoades, J. C. Nelson, I. Nwabudike, S. K. Yu, I. G. McLachlan, G. K. Madan, E. Abebe, J. R. Powers, D. A. Colón-Ramos, S. W. Flavell, ASICs mediate food responses in an enteric serotonergic neuron that controls foraging behaviors. *Cell* **176**, 85–97.e14 (2019).

24. J. P. Brandt, N. Ringstad, Toll-like receptor signaling promotes development and function of sensory neurons required for a C. elegans pathogen-avoidance behavior. *Curr. Biol.* **25**, 2228–2237 (2015).

25. E. A. Hallem, W. C. Spencer, R. D. McWhirter, G. Zeller, S. R. Henz, G. Rätsch, D. M. Miller III, H. R. Horvitz, P. W. Sternberg, N. Ringstad, Receptor-type guanylate cyclase is required for carbon dioxide sensation by Caenorhabditis elegans. *Proc. Natl. Acad. Sci. U.S.A.* **108**, 254–259 (2011).

26. N. Ringstad, H. R. Horvitz, FMRFamide neuropeptides and acetylcholine synergistically inhibit egg-laying by C. elegans. *Nat. Neurosci.* **11**, 1168–1176 (2008).

27. L. Emtage, S. Aziz-Zaman, O. Padovan-Merhar, H. R. Horvitz, C. Fang-Yen, N. Ringstad, IRK-1 potassium channels mediate peptidergic inhibition of Caenorhabditis elegans serotonin neurons via a G_o signaling pathway. *J. Neurosci.* **32**, 16285–16295 (2012).

28. K. E. Zang, E. Ho, N. Ringstad, Inhibitory peptidergic modulation of *C. elegans* serotonin neurons is gated by T-type calcium channels. *eLife* **6**, e22771 (2017).

29. K. E. J. Rödström, A. K. Kiper, W. Zhang, S. Rinné, A. C. W. Pike, M. Goldstein, L. J. Conrad, M. Delbeck, M. G. Hahn, H. Meier, M. Platzk, A. Quigley, D. Speedman, L. Shrestha, S. M. M. Mukhopadhyay, N. A. Burgess-Brown, S. J. Tucker, T. Müller, N. Decher, E. P. Carpenter, A lower X-gate in TASK channels traps inhibitors within the vestibule. *Nature* **582**, 443–447 (2020).

30. J. Sörmann, M. Schewe, P. Proks, T. Jouen-Tachoire, S. Rao, E. B. Riel, K. E. Agre, A. Begtrup, J. Dean, M. Descartes, J. Fischer, A. Gardham, C. Lahner, P. R. Mark, S. Muppidi, P. N. Pichurin, J. Porrmann, J. Schallner, K. Smith, V. Straub, P. Vasudevan, R. Willaert, E. P. Carpenter, K. E. J. Rödström, M. G. Hahn, T. Müller, T. Baukrowitz, M. E. Hurles, C. F. Wright, S. J. Tucker, Gain-of-function mutations in *KCNK3* cause a developmental disorder with sleep apnea. *Nat. Genet.* **54**, 1534–1543 (2022).

31. Q. Zhang, J. Fu, S. Zhang, P. Guo, S. Liu, J. Shen, J. Guo, H. Yang, ‘C-type’ closed state and gating mechanisms of K2P channels revealed by conformational changes of the TREK-1 channel. *J. Mol. Cell Biol.* **14**, mjac002 (2022).

32. H. Wang, H. Park, J. Liu, P. W. Sternberg, An efficient genome editing strategy to generate putative null mutants in Caenorhabditis elegans using CRISPR/Cas9. *G3* **8**, 3607–3616 (2018).

33. C. Trent, N. Tsung, H. R. Horvitz, Egg-laying defective mutants of the nematode Caenorhabditis elegans. *Genetics* **104**, 619–647 (1983).

34. S. A. Daniels, M. Ailion, J. H. Thomas, P. Sengupta, egl-4 acts through a transforming growth factor-beta/SMAD pathway in Caenorhabditis elegans to regulate multiple neuronal circuits in response to sensory cues. *Genetics* **156**, 123–141 (2000).

35. J. G. White, E. Southgate, J. N. Thomson, S. Brenner, The structure of the ventral nerve cord of Caenorhabditis elegans. *Philos. Trans. R. Soc. Lond. B Biol. Sci.* **275**, 327–348 (1976).

36. A. Oranth, C. Schultheis, O. Tolstenkov, K. Erbguth, J. Nagpal, D. Hain, M. Brauner, S. Wabnig, W. Steuer Costa, R. D. McWhirter, S. Zels, S. Palumbos, D. M. Miller Iii, I. Beets, A. Gottschalk, Food sensation modulates locomotion by dopamine and neuropeptide signaling in a distributed neuronal network. *Neuron* **100**, 1414–1428.e10 (2018).

37. L. B. Horowitz, J. P. Brandt, N. Ringstad, Repression of an activity-dependent autocrine insulin signal is required for sensory neuron development in *C. elegans*. *Development* **146**, dev182873 (2019).

38. S. R. Taylor, G. Santpere, A. Weinreb, A. Barrett, M. B. Reilly, C. Xu, E. Varol, P. Oikonomou, L. Glenwinkel, R. McWhirter, A. Poff, M. Basavaraju, I. Rafi, E. Yemini, S. J. Cook, A. Abrams, B. Vidal, C. Cros, S. Tavazoie, N. Sestan, M. Hammarlund, O. Hobert, D. M. Miller III, Molecular topography of an entire nervous system. *Cell* **184**, 4329–4347.e23 (2021).

39. J. W. Much, D. J. Slade, K. Klampert, G. Garriga, B. Wightman, The fax-1 nuclear hormone receptor regulates axon pathfinding and neurotransmitter expression. *Development* **127**, 703–712 (2000).

40. N. Pokala, Q. Liu, A. Gordus, C. I. Bargmann, Inducible and titratable silencing of Caenorhabditis elegans neurons in vivo with histamine-gated chloride channels. *Proc. Natl. Acad. Sci. U.S.A.* **111**, 2770–2775 (2014).

41. H. Ji, A. D. Fouad, Z. Li, A. Ruba, C. Fang-Yen, A proprioceptive feedback circuit drives *Caenorhabditis elegans* locomotor adaptation through dopamine signaling. *Proc. Natl. Acad. Sci. U.S.A.* **120**, e2219341120 (2023).

42. N. Cermak, S. K. Yu, R. Clark, Y. C. Huang, S. N. Baskoylu, S. W. Flavell, Whole-organism behavioral profiling reveals a role for dopamine in state-dependent motor program coupling in *C. elegans*. *eLife* **9**, e57093 (2020).

43. E. G. Govorunova, O. A. Sineshchekov, R. Janz, X. Liu, J. L. Spudich, Natural light-gated anion channels: A family of microbial rhodopsins for advanced optogenetics. *Science* **349**, 647–650 (2015).

44. N. C. Klapoetke, Y. Murata, S. S. Kim, S. R. Pulver, A. Birdsey-Benson, Y. K. Cho, T. K. Morimoto, A. S. Chuong, E. J. Carpenter, Z. Tian, J. Wang, Y. Xie, Z. Yan, Y. Zhang, B. Y. Chow, B. Surek, M. Melkonian, V. Jayaraman, M. Constantine-Paton, G. K.-S. Wong, E. S.

Boyden, Independent optical excitation of distinct neural populations. *Nat. Methods* **11**, 338–346 (2014).

45. O. Hobert, L. Glenwinkel, J. White, Revisiting neuronal cell type classification in Caenorhabditis elegans. *Curr. Biol.* **26**, R1197–R1203 (2016).

46. C. Wang, B. Vidal, S. Sural, C. Loer, G. R. Aguilar, D. M. Merritt, I. A. Toker, M. C. Vogt, C. C. Cros, O. Hobert, A neurotransmitter atlas of *C. elegans* males and hermaphrodites. *eLife* **13**, RP95402 (2024).

47. L. Ripoll-Sanchez, J. Watteyne, H. Sun, R. Fernandez, S. R. Taylor, A. Weinreb, B. L. Bentley, M. Hammarlund, D. M. Miller III, O. Hobert, I. Beets, P. E. Vertes, W. R. Schafer, The neuropeptidergic connectome of *C. elegans*. *Neuron* **111**, 3570–3589.e5 (2023).

48. S. T. Sweeney, K. Broadie, J. Keane, H. Niemann, C. J. O'Kane, Targeted expression of tetanus toxin light chain in Drosophila specifically eliminates synaptic transmission and causes behavioral defects. *Neuron* **14**, 341–351 (1995).

49. S. Speese, M. Petrie, K. Schuske, M. Ailion, K. Ann, K. Iwasaki, E. M. Jorgensen, T. F. J. Martin, UNC-31 (CAPS) is required for dense-core vesicle but not synaptic vesicle exocytosis in Caenorhabditis elegans. *J. Neurosci.* **27**, 6150–6162 (2007).

50. D. Sieburth, J. M. Madison, J. M. Kaplan, PKC-1 regulates secretion of neuropeptides. *Nat. Neurosci.* **10**, 49–57 (2007).

51. I. Buntschuh, D. A. Raps, I. Joseph, C. Reid, A. Chait, R. Totanes, M. Sawh, C. Li, FLP-1 neuropeptides modulate sensory and motor circuits in the nematode Caenorhabditis elegans. *PLOS ONE* **13**, e0189320 (2018).

52. J. Marquina-Solis, L. Feng, E. Vandewyer, I. Beets, J. Hawk, D. A. Colon-Ramos, J. Yu, B. W. Fox, F. C. Schroeder, C. I. Bargmann, Antagonism between neuropeptides and monoamines in a distributed circuit for pathogen avoidance. *Cell Rep.* **43**, 114042 (2024).

53. Y. L. Chew, L. J. Grundy, A. E. X. Brown, I. Beets, W. R. Schafer, Neuropeptides encoded by *nlp-49* modulate locomotion, arousal and egg-laying behaviours in *Caenorhabditis elegans* via the receptor SEB-3. *Philos. Trans. R. Soc. Lond. B Biol. Sci.* **373**, 20170368 (2018).

54. T. Janssen, S. J. Husson, M. Lindemans, I. Mertens, S. Rademakers, K. Ver Donck, J. Geysen, G. Jansen, L. Schoofs, Functional characterization of three G protein-coupled receptors for pigment dispersing factors in Caenorhabditis elegans. *J. Biol. Chem.* **283**, 15241–15249 (2008).

55. I. Beets, S. Zels, E. Vandewyer, J. Demeulemeester, J. Caers, E. Baytemur, A. Courtney, L. Golinelli, İ. Hasakioğulları, W. R. Schafer, P. E. Vértes, O. Mirabeau, L. Schoofs, System-wide mapping of peptide-GPCR interactions in *C. elegans*. *Cell Rep.* **42**, 113058 (2023).

56. A. Barrios, R. Ghosh, C. Fang, S. W. Emmons, M. M. Barr, PDF-1 neuropeptide signaling modulates a neural circuit for mate-searching behavior in C. elegans. *Nat. Neurosci.* **15**, 1675–1682 (2012).

57. S. W. Flavell, N. Pokala, E. Z. Macosko, D. R. Albrecht, J. Larsch, C. I. Bargmann, Serotonin and the neuropeptide PDF initiate and extend opposing behavioral states in *C. elegans*. *Cell* **154**, 1023–1035 (2013).

58. F. Schnütgen, N. Doerflinger, C. Calléja, O. Wendling, P. Chambon, N. B. Ghyselinck, A directional strategy for monitoring Cre-mediated recombination at the cellular level in the mouse. *Nat. Biotechnol.* **21**, 562–565 (2003).

59. O. Hobert, I. Mori, Y. Yamashita, H. Honda, Y. Ohshima, Y. Liu, G. Ruvkun, Regulation of interneuron function in the *C. elegans* thermoregulatory pathway by the *ttx-3* LIM homeobox gene. *Neuron* **19**, 345–357 (1997).

60. O. T. Shafer, D. J. Kim, R. Dunbar-Yaffe, V. O. Nikolaev, M. J. Lohse, P. H. Taghert, Widespread receptivity to neuropeptide PDF throughout the neuronal circadian clock network of Drosophila revealed by real-time cyclic AMP imaging. *Neuron* **58**, 223–237 (2008).

61. C. G. Vecsey, N. Pírez, L. C. Griffith, The Drosophila neuropeptides PDF and sNPF have opposing electrophysiological and molecular effects on central neurons. *J. Neurophysiol.* **111**, 1033–1045 (2014).

62. L. Pereira, P. Kratsios, E. Serrano-Saiz, H. Sheftel, A. E. Mayo, D. H. Hall, J. G. White, B. LeBoeuf, L. R. Garcia, U. Alon, O. Hobert, A cellular and regulatory map of the cholinergic nervous system of C. elegans. *eLife* **4**, e12432 (2015).

63. R. Li, Y. Xu, X. Wen, Y.-H. Chen, P.-Z. Wang, J.-L. Zhao, P.-P. Wu, J.-J. Wu, H. Liu, J.-H. Huang, S.-J. Li, Z.-X. Wu, GCY-20 signaling controls suppression of Caenorhabditis elegans egg laying by moderate cold. *Cell Rep.* **43**, 113708 (2024).

64. A. Klaus, J. Alves da Silva, R. M. Costa, What, if, and when to move: Basal ganglia circuits and self-paced action initiation. *Annu. Rev. Neurosci.* **42**, 459–483 (2019).

65. J. J. Letzkus, S. B. Wolff, A. Lüthi, Disinhibition, a circuit mechanism for associative learning and memory. *Neuron* **88**, 264–276 (2015).

66. E. Honoré, The neuronal background K2P channels: Focus on TREK1. *Nat. Rev. Neurosci.* **8**, 251–261 (2007).

67. S. A. Siegelbaum, J. S. Camardo, E. R. Kandel, Serotonin and cyclic AMP close single K^+ channels in *Aplysia* sensory neurones. *Nature* **299**, 413–417 (1982).

68. A. J. Patel, E. Honoré, F. Maingret, F. Lesage, M. Fink, F. Duprat, M. Lazdunski, A mammalian two pore domain mechano-gated S-like K^+ channel. *EMBO J.* **17**, 4283–4290 (1998).

69. D. Pimentel, J. M. Donlea, C. B. Talbot, S. M. Song, A. J. F. Thurston, G. Miesenböck, Operation of a homeostatic sleep switch. *Nature* **536**, 333–337 (2016).

70. D. C. Gottschling, F. Döring, K. Lüersen, Locomotion behavior is affected by the $G\alpha_S$ pathway and the two-pore-domain K^+ channel TWK-7 interacting in GABAergic motor neurons in *Caenorhabditis elegans*. *Genetics* **206**, 283–297 (2017).

71. F. Duprat, I. Lauritzen, A. Patel, E. Honoré, The TASK background K_{2P} channels: Chemo- and nutrient sensors. *Trends Neurosci.* **30**, 573–580 (2007).

72. T. Wu, F. Duan, W. Yang, H. Liu, A. Caballero, D. A. Fernandes de Abreu, A. R. Dar, J. Alcedo, Q. Ch'ng, R. A. Butcher, Y. Zhang, Pheromones modulate learning by regulating the balanced signals of two insulin-like peptides. *Neuron* **104**, 1095–1109.e5 (2019).

73. S. Brenner, The genetics of Caenorhabditis elegans. *Genetics* **77**, 71–94 (1974).

74. G. van der Auwera, B. D. O'Connor, *Genomics in the Cloud : Using Docker, GATK, and WDL in Terra* (O'Reilly Media, ed. 1, 2020), p. 24, 467 pp.

75. P. Cingolani, A. Platts, L. L. Wang, M. Coon, T. Nguyen, L. Wang, S. J. Land, X. Lu, D. M. Ruden, A program for annotating and predicting the effects of single nucleotide polymorphisms, SnpEff: SNPs in the genome of Drosophila melanogaster strain w1118; iso-2; iso-3. *Fly* **6**, 80–92 (2012).

76. P. W. Sternberg, K. Van Auken, Q. Wang, A. Wright, K. Yook, M. Zarowiecki, V. Arnaboldi, A. Becerra, S. Brown, S. Cain, J. Chan, W. J. Chen, J. Cho, P. Davis, S. Diamantakis, S. Dyer, D. Grigoriadis, C. A. Grove, T. Harris, K. Howe, R. Kishore, R. Lee, I. Longden, M. Luypaert, H. M. Muller, P. Nuin, M. Quinton-Tulloch, D. Raciti, T. Schedl, G. Schindelman, L. Stein, WormBase 2024: Status and transitioning to Alliance infrastructure. *Genetics* **227**, iyae050 (2024).

77. C. C. Mello, J. M. Kramer, D. Stinchcomb, V. Ambros, Efficient gene transfer in C.elegans: Extrachromosomal maintenance and integration of transforming sequences. *EMBO J.* **10**, 3959–3970 (1991).

78. A. Paix, A. Folkmann, D. Rasoloson, G. Seydoux, High efficiency, homology-directed genome editing in Caenorhabditis elegans using CRISPR-Cas9 ribonucleoprotein complexes. *Genetics* **201**, 47–54 (2015).

79. S. Berg, D. Kutra, T. Kroeger, C. N. Straehle, B. X. Kausler, C. Haubold, M. Schiegg, J. Ales, T. Beier, M. Rudy, K. Eren, J. I. Cervantes, B. Xu, F. Beuttenmueller, A. Wolny, C. Zhang, U.

Koethe, F. A. Hamprecht, A. Kreshuk, ilastik: Interactive machine learning for (bio)image analysis. *Nat. Methods* **16**, 1226–1232 (2019).

80. J. Schindelin, I. Arganda-Carreras, E. Frise, V. Kaynig, M. Longair, T. Pietzsch, S. Preibisch, C. Rueden, S. Saalfeld, B. Schmid, J. Y. Tinevez, D. J. White, V. Hartenstein, K. Eliceiri, P. Tomancak, A. Cardona, Fiji: An open-source platform for biological-image analysis. *Nat. Methods* **9**, 676–682 (2012).

81. J. S. Duerr, Immunostainings in nervous system development of the nematode C. elegans. *Methods Mol. Biol.* **2047**, 293–310 (2020).

82. S. L. Edwards, N. K. Charlie, M. C. Milfort, B. S. Brown, C. N. Gravlin, J. E. Knecht, K. G. Miller, A novel molecular solution for ultraviolet light detection in Caenorhabditis elegans. *PLoS Biol.* **6**, e198 (2008).

83. J. T. Robinson, H. Thorvaldsdottir, W. Winckler, M. Guttman, E. S. Lander, G. Getz, J. P. Mesirov, Integrative genomics viewer. *Nat. Biotechnol.* **29**, 24–26 (2011).

84. M. I. Love, W. Huber, S. Anders, Moderated estimation of fold change and dispersion for RNA-seq data with DESeq2. *Genome Biol.* **15**, 550 (2014).

85. J. Jumper, R. Evans, A. Pritzel, T. Green, M. Figurnov, O. Ronneberger, K. Tunyasuvunakool, R. Bates, A. Žídek, A. Potapenko, A. Bridgland, C. Meyer, S. A. A. Kohl, A. J. Ballard, A. Cowie, B. Romera-Paredes, S. Nikolov, R. Jain, J. Adler, T. Back, S. Petersen, D. Reiman, E. Clancy, M. Zielinski, M. Steinegger, M. Pacholska, T. Berghammer, S. Bodenstein, D. Silver, O. Vinyals, A. W. Senior, K. Kavukcuoglu, P. Kohli, D. Hassabis, Highly accurate protein structure prediction with AlphaFold. *Nature* **596**, 583–589 (2021).
